# Additive Manufacturing Strategies for Personalized Drug Delivery Systems and Medical Devices: Fused Filament Fabrication and Semi Solid Extrusion

**DOI:** 10.3390/molecules27092784

**Published:** 2022-04-27

**Authors:** Giulia Auriemma, Carmela Tommasino, Giovanni Falcone, Tiziana Esposito, Carla Sardo, Rita Patrizia Aquino

**Affiliations:** 1 Department of Pharmacy, University of Salerno, Via Giovanni Paolo II 132, I-84084 Fisciano, Italy; gauriemma@unisa.it (G.A.); ctommasino@unisa.it (C.T.); gifalcone@unisa.it (G.F.); tesposito@unisa.it (T.E.); csardo@unisa.it (C.S.); 2 PhD Program in Drug Discovery and Development, University of Salerno, Via Giovanni Paolo II 132, I-84084 Fisciano, Italy

**Keywords:** additive manufacturing, 3D-Printing, rapid prototyping, FFF, SSE, personalized therapy, customized DDS, medical devices

## Abstract

Novel additive manufacturing (AM) techniques and particularly 3D printing (3DP) have achieved a decade of success in pharmaceutical and biomedical fields. Highly innovative personalized therapeutical solutions may be designed and manufactured through a layer-by-layer approach starting from a digital model realized according to the needs of a specific patient or a patient group. The combination of patient-tailored drug dose, dosage, or diagnostic form (shape and size) and drug release adjustment has the potential to ensure the optimal patient therapy. Among the different 3D printing techniques, extrusion-based technologies, such as fused filament fabrication (FFF) and semi solid extrusion (SSE), are the most investigated for their high versatility, precision, feasibility, and cheapness. This review provides an overview on different 3DP techniques to produce personalized drug delivery systems and medical devices, highlighting, for each method, the critical printing process parameters, the main starting materials, as well as advantages and limitations. Furthermore, the recent developments of fused filament fabrication and semi solid extrusion 3DP are discussed. In this regard, the current state of the art, based on a detailed literature survey of the different 3D products printed via extrusion-based techniques, envisioning future directions in the clinical applications and diffusion of such systems, is summarized.

## 1. Introduction

In the last few years, the interest in three-dimensional printing (3DP) in the scientific world, and particularly in pharmaceutical and medical research, has grown exponentially. In fact, the number of scientific papers recorded in the Web of Science Core Collection containing the term “3D printing” in the title increased from 57 in 2012 to 4623 in 2021. In addition, the number of citations of these papers in the same period grew from 23 to 28,438. Narrowing the searching results to the pharmacy/pharmacology category, no result was found in 2012, whereas 553 records were found up to 2021. In the light of this analysis, it is possible to say with certainty that 3DP represents today one of the fastest developing technologies in the healthcare field.

The term 3D printing is defined by International Standard Organization (ISO) as the “fabrication of objects through the deposition of a material using a print head, nozzle, or another printer technology” [1]. This includes a wide variety of techniques able to precisely produce freeform solid objects of a high degree of complexity starting from digital models created with computer aided design (CAD), ensuring great fidelity, reproducibility, and cost-effectiveness [2]. The application of 3DP in the scientific area has become more and more relevant since 2012 [1]. In the pharmaceutical field, a great impact was made with the approval by the FDA in August 2015 of the first 3D-printed drug product, Spritam^®^. This antiepileptic oro dispersible tablet (ODT) loaded with levetiracetam [3] was obtained by Aprecia Pharmaceuticals by using Massachusetts Institute of Technology (MIT) patented Zip-Dose Technology [4,5]. This 3DP dosage form has a highly porous structure with dose strengths up to 1000 mg that could not be achieved with traditional manufacturing. Thanks to its porous structure, Spritam^®^ is able to disintegrate and dissolve within few seconds upon contact with saliva, helping both elderly and young patients suffering from trouble swallowing pills, known as dysphagia [6,7].

**Figure 1:**
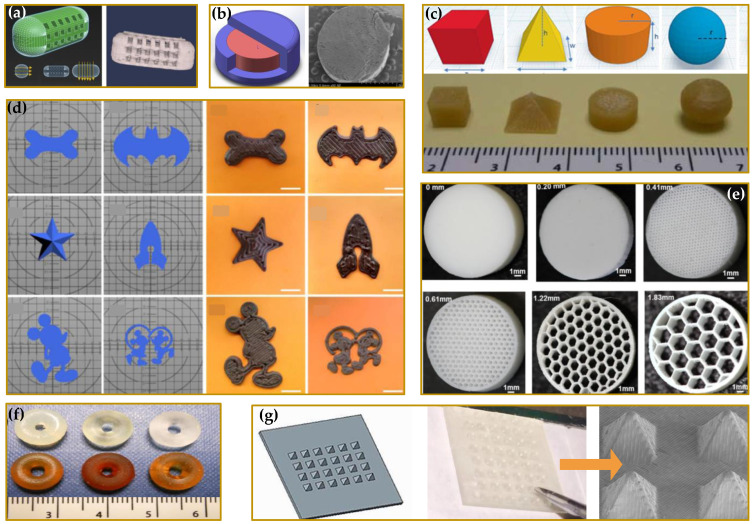
Examples of 3D printed products for drug delivery. (**a**) Channeled tablet. Reprinted with permission from reference [35]; copyright (2017) Elsevier B.V. (**b**) Duo Tablet. Reprinted with permission from reference [36]; copyright (2017) Elsevier B.V. (**c**) Cube, pyramid, cylinder and sphere-shaped tablets. Reprinted with permission from reference [37]; copyright (2015) Elsevier B.V. (**d**) Chewable chocolate-based oral dosage forms. Reprinted with permission from reference [38]; copyright (2020) Elsevier B.V. (**e**) Tablets with honeycomb architectures. Reprinted from reference [39]; copyright (2017). (**f**) Donut-shaped tablets. Reprinted with permission from reference [40]; copyright (2016) Elsevier B.V. (**g**) Microneedle patch. Reprinted with permission from reference [41]. copyright (2018) Elsevier B.V.

**Figure 2:**
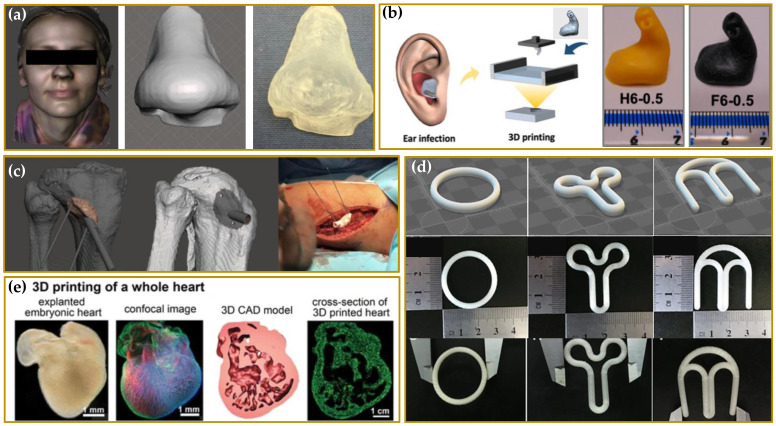
Examples of 3D printed products for biomedical applications. (**a**) Nose-shaped device. Reprinted with permission from reference [42]; copyright (2016) Elsevier B.V. (**b**) Anti-biofilm hearing aids. Reprinted with permission from reference [43]; copyright (2021) Elsevier B.V. (**c**) Guide used during a surgery for tibial plateau fracture. Reprinted from reference [44]; copyright (2021). (**d**) Vaginal rings. Reprinted with permission from reference [45]; copyright (2018) Elsevier B.V. (**e**) 3D printed heart. Reprinted from reference [46]; copyright (2016).

The impact of FDA approval has caused a really fast increase in the number of studies and scientific researches on the 3DP technologies with noteworthy results mainly for the development of tablets [8,9,10,11,12,13,14,15,16], capsules [17,18], orodispersible films [19,20,21,22,23,24], and medical devices [25,26,27,28,29]. Such achievements have brought to the light the real potential of 3DP as an effective tool to realize personalized therapeutic solutions fitting specific patient needs [30,31]. In Figure 1 and Figure 2, just some examples of the recently developed 3D printed customized products are reported with a great variety of structures, shapes, and layers. The remarkable results achieved have certainly not left unmoved the big pharmaceutical companies. The 3D-printed pharmaceuticals market was valued at $175.19 million in 2020 and anticipated to grow to $285.17 million by 2025, representing a significant opportunity to companies able to capitalize on its benefits and overcome its challenges [32]. Several big pharmaceutical companies have accepted the challenge to explore the emerging 3DP technologies, and possibly to integrate them into their workflows, with investments of millions of dollars. In 2020, the company Merck announced plans to work with EOS Group Company ACMC to produce 3D printed tablets first for clinical trials, then later for commercial manufacturing. In the same year, Aprecia announced their long term collaboration with R&D firm Battelle to expand its capabilities within 3D printed pharmaceuticals and advance its 3D printing equipment from clinical supply to commercial scale. Between 2020 and 2021, Triastek raised millions of dollars in funding to support the ongoing development of T19, its first 3D printed product approved by the FDA as an Investigational New Drug (IND), and expand the 3D printed drug product pipeline. T19 is a chronotherapeutic drug delivery system produced by melt extrusion deposition (MED) technology, accepted in April 2021 within the FDA’s Emerging Technology Program (ETP). The company has announced for T19 a New Drug Application (NDA) submission in 2023, but the scenario is rapidly evolving. In fact, the second product of Triastek, T20, received positive pre-IND feedback from FDA in March 2021, and an IND application submission for T20 is planned for the end of the year [33,34].

Although 3DP has been only recently explored for the manufacturing of personalized drug delivery systems (PDDS), such technology is well established in various biomedical areas for the creation, e.g., of customized prosthesis, orthopedic implants and anatomical models, surgical instrumentations, etc. Just think, for example, that, since 2001 Sonova has been able to produce hundreds of thousands of custom-made hearing aids every year [47] by 3DP technology. The Sonova model is a significant example of the customized manufacturing of a medical device and how personalization can change, or rather, has changed the hearing aid industry for better. A lot has been done in this specific field, but a lot remains to be done in other biomedical sectors as well as in the pharmaceutical one [48,49].

The aim of the present review is to provide an overview of the opportunities and challenges of 3DP technologies for pharmaceutical and biomedical applications, more specifically addressing the main driving force in the future developments of both 3DP techniques and products (a). A brief account of the basic aspects of a generic 3D printing process (b) is given to discuss their bridge with the main 3DP methods exploitable to produce complex and customizable dosage forms and/or medical devices (c), and related advantages and limitations. The most used and versatile extrusion-based 3DP techniques, FFF and SSE, and their main applications are finally discussed (d).

## 2. Driving Force in the Developing of 3DP Medicines and Medical Devices

The need for therapeutic approaches specific to the individual, the increasing demand for complex drug-eluting products, medical devices, and advanced drug-device combination products, as well as the growing request of on-demand manufacturing, are the main reasons strongly promoting the use of 3DP technology in both pharmaceutical and biomedical fields.

**Figure 3:**
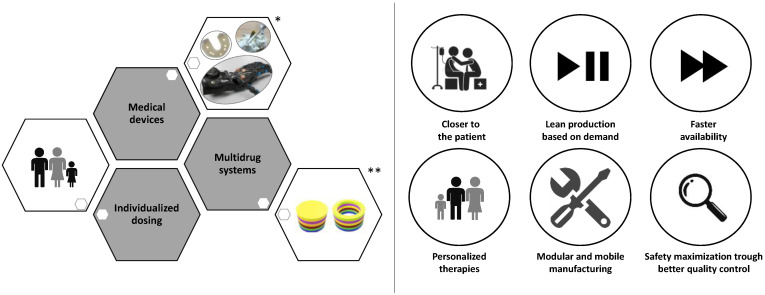
Personalized medical approach. * Images adapted from reference [55]. ** Images adapted from reference [56].

**Figure 4:**
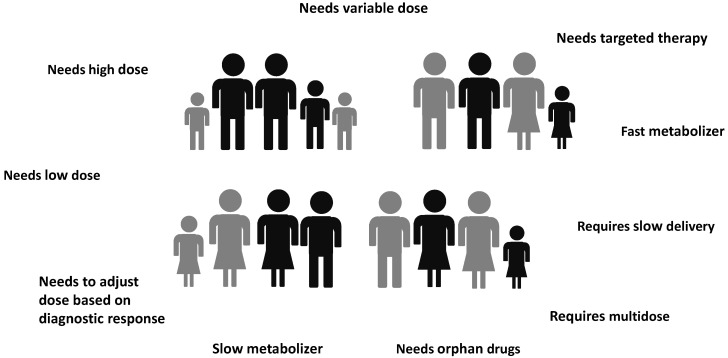
Possible advantages deriving from a personalized therapy.

Personalized medicine, also indicated as precision medicine, is a medical approach that separates people into different groups based on individual needs. Personalized medicine takes into account the genetic profile, lifestyle, environment, weight, sex, and age as well as other specific patient needs. A personalized medical approach could be represented by a medical decision or practice, an intervention, and/or a product being tailored to the individual patient based on its predicted response or risk of disease [50,51]. From this point of view, 3D printing possesses the great potential [52,53,54] of linking the patient, with all its real needs, to tailor-made medicines and treatments (Figure 3 and Figure 4).

3DP presents the possibility to easily develop patient-centered dosage forms, answering to the need to deliver “the right drug at the right dose and at the right time” [49], e.g., age-appropriate dosing for pediatrics and geriatrics sub-populations [57,58], multiple drug administration in polypharmacy practice [59], and innovative dosage forms, enhancing patient compliance and adherence to treatment with respect to conventional solid dosage forms [56]. Besides the proVazper drug dosage, with this approach, it is possible to produce controlled drug delivery systems with tailored drug release profile and even, multiple drug content. A “customized polypill” is a solid oral dosage [60] form 3D-printed in a complex construct of layers, capable of satisfying more than one therapeutic need at the same time. A “polypill” can be realized as a multi-drug pill, carrying and delivering a combination of drugs with various controlled release mechanisms to treat multiple diseases at once, or as a multi-dose pill, charged with different doses of the same drug to be delivered at different times. In addition, the possibility to guarantee a precise drug loading within the 3D printed structure increases, in general, the safety of all the selected drugs, above all of those characterized by a narrow therapeutic window requiring an exact dosing.

3DP continues to revolutionize the medical device landscape, continually providing higher performing patient specific anatomic models, such as prostheses [61,62,63,64] and dental [65,66] or orthopedic implants [67,68]. Its application is also growing in tissue engineering (TE) and regenerative medicine (RM) for the manufacturing of three-dimensional scaffolds acting as biological substitutes of damaged tissues or organs [69,70,71].

Another exciting opportunity to become a focus area for research and development lies in the powerful combination of drug eluting products and medical devices. Advanced drug-eluting devices providing significant and unique benefits to patients over conventional treatments have been recently developed by different 3DP technologies in the form of antibiotic and chemotherapeutic catheters [72], antimicrobial stents [73] and implants [74,75,76], and even anti-biofilm hearing aids [43] or anti-glaucoma contact lenses [77].

**Figure 5:**
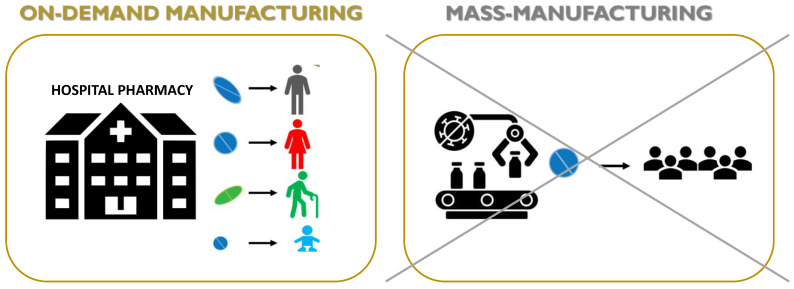
On-demand manufacturing (customized products) vs. mass manufacturing (traditional medicines, one-size-fits-all). The new scenario opened by 3DP technology.

3DP offers a forward-looking view for producing as well as dispensing medicines, moving the attention from traditional mass manufacturing to on demand manufacturing, and thus from centralized towards decentralized facilities (Figure 5). Therefore, based on a patient specific prescription from their doctor, a customized medicinal product with complex geometry and architecture, charged with multiple doses of a specific drug, or even loaded with multiple drugs, can be designed via CAD and produced, when needed, by a 3D printer. Potentially, a hospital, clinic, community pharmacy, or even the patient’s home, may be engaged in the pharmaceutical compounding/production [1]. Recently, Beer et al. investigated the possible implementation of 3DP technologies in the European pharmaceutical system. Among the various scenarios suggested (namely 3D printers in hospital pharmacies, community pharmacies, compounding facilities, patients’ homes, and industry), those involving 3D printing in patients’ homes was presented as the most futuristic, whereas printing at hospitals and pharmacies where other routine compounding is already taking place were shown as more realistic [48].

3D manufacturing may be a good alternative to the compounding practice in hospital settings when a precise dosage, small batches for clinical studies, orphan drugs, and expensive oncology medical preparations are needed. In this case, 3DP appears cost- and material-saving, produces a smaller footprint, and may increase precision dosing, safety, and benefits to the patients. The overall printing process (materials’ control, feedstock setting-up, printing, cleaning etc.) could be slower than the conventional compounding based on simple operations (such as weighing, grinding, mixing, diluting, cleaning), but it is still worth exploring. This great potential has allowed for the creation of point-of-care (PoC) 3D printing centers, which blur the line between the healthcare provider, medical center, and device manufacturer, creating regulatory ambiguity [78]. Currently, the FDA remains undecided about the best way to regulate PoC printing centers. However, they have recently begun working with stakeholders (including engineers, the medical device industry, various 3DP interesting workgroups, PoC manufacturing centers, physicians, and surgeons) through a webinar series hosted by the American Society of Mechanical Engineers (ASME) to develop a regulatory framework [79], following the way opened by the UK agency MHRA [80].

There is a fundamental difference when discussing printing in the context of small-scale compounding in the hospital and pharmacy setting or large-scale manufacturing in the industry setting. In the industrial setting, the main benefits deriving from decentralizing pharmaceutical manufacture are the following [1,30]:

1. Reduced length and cost of transport and storage [81].
2. Quick and real-time responses to patient and market needs due to the possibility to rapidly produce small batches of complex formulations with unique geometries and, furthermore, the concept of digital dispensing in hard-to-reach areas or developing countries [82].
3. Reduced waste and hence reduced costs of developing and dosing due to a precise spatial control over the deposition of materials, limiting the amounts of API (active pharmaceutical ingredient) and excipients in comparison to conventional technologies [83].

Despite these benefits, there are several technical and regulatory challenges that need to be overcome before 3D printing may be widely used for pharmaceutical applications in clinical practice, as shown by only one FDA approval of a 3D printed drug on the market [3], and one on its way [33]. In the industrial setting, a 3D printed product must comply with the current manufacturing and control standards for medical products and devices, specifically the well-stablished Good Manufacturing Practices (GMP). However, 3DP manufacturing has many more issues involving design and production, raw material storage and transport, quality control, risk of counterfeit production, etc. In this regard, significant progress has been made for medical devices, ranging from surgical planning tool to custom surgical devices [84]. In the last few years, various papers have focused on the analysis of critical process parameters as well as product quality attributes and performance criteria, which must be addressed considering the industrial system of quality assurance to guarantee the fulfilment of regulatory standards [84,85]. Implantable medical devices require, for example, along with the careful characterization of starting materials as well as the optimization of 3D printing parameters, additional considerations about cleaning, finishing, and sterilization procedures [86]. In the attempt to allow the overcoming of such hurdles, the FDA released in December 2017 guidance detailing the technical considerations for additive manufactured medical devices, from software and hardware requirements, quality control, up to process validation procedures [87]. As the FDA document highlighted, due to the variability of additive manufacturing methods, there is no possibility to give one universal set of 3DP guidelines. It seems that every single printing method needs different equipment, starting materials, post-processing treatments, laboratories, and hence separate regulatory requirements [84]. The situation is complicated even more with the inclusion of one or more APIs, as it happens for multi-drug medicinal products which must considered possible incompatibilities and APIs’ stability problems. Generally, requirements for drug-eluting products are more demanding than those for medical devices and this is the key reason why there are still no regulations. However, to fully understand the real motivation causing the actual regulatory difficulties, it is essential to analyze this new production approach from a strictly technical point of view.

## 3. 3D Printing: Technical Aspects

### 3.1. What Is 3D-Printing from a Technical Point of View?

**Figure 6:**
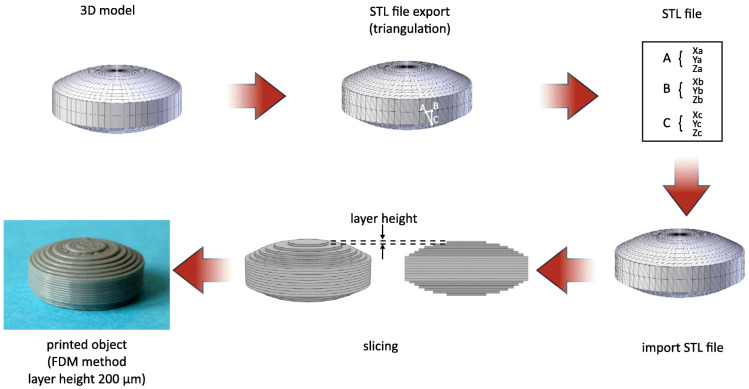
3D-Printing phases to realize a printlet by FDM method. Reprinted from reference [1].

Technically, 3DP can be defined as a layer-by-layer production of 3D objects from digital designs. This technology belongs to the so-called additive manufacturing (AM) processes because the object is produced through the layer-by-layer deposition of starting materials using a print head, nozzle, or another printer technology. Even the term rapid prototyping (RP) is often used to define 3DP since it generally refers to all techniques able to construct objects from digital models created with CAD software. Therefore, additive manufacturing is mainly referred to as the productive step, involving rapid prototyping through the whole procedure [88]. Despite of the diversity of 3DP methods, all exploit a CAD-CAM system. The process starts from the design of a digital model using CAD software; the latter is converted in a STL. file, a machine-readable format which describes the external surface of a 3D model. After this step, the STL. file is imported to the printer software (CAM software) that, through a slicing process, generates the layers which will be printed in an additive way by the printer. The height of the printed layer essentially influences the quality of the printed object as well as printing time. In addition, a specific post-processing step could be required to get the final product, based on the specific 3DP method selected. Figure 6 shows in a detailed way the main steps involved during the 3DP of a printlet (3D printed tablet) using fused deposition modeling as the productive technology.

### 3.2. 3D Printing Methods

**Figure 7:**
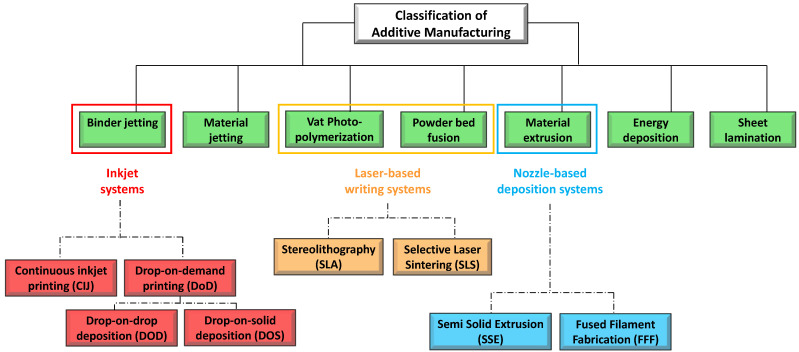
Schematic view of additive manufacturing processes according to ISO/ASTM 52,900:2021 classification [89,90], with in evidence the main AM methods applied in pharmaceutical and biomedical field [54,91].

Various and very different 3D printing techniques exist, depending on the specific technology used. All of them fall within the common definition of solid freeform fabrication (SFF) aiming to focus on the possibility to produce solid free forms with a complex and well-defined architecture. According to the American Society for Testing and Materials (ASTM F2792-12a), AM processes can be classified in seven categories, namely: (1) material jetting, (2) binder jetting, (3) vat photopolymerization, (4) powder bed fusion, (5) material extrusion, (6) energy deposition, and (7) sheet lamination [89] (Figure 7). Further AM classifications may be done based on the physical state of the starting material used to form the product (solid, liquid, and powder-based processes), or even the medium used for its processing (laser beam, ultraviolet rays, thermal means, etc.) [90]. A commonly accepted classification of the different 3DP systems used for pharmaceutical and medical applications is based on three main groups, namely:

1. Printing based **ink-jet systems**
2. **Laser**-based writing systems,
3. **Nozzle**-based deposition systems [54,91].

#### 3.2.1. Ink-Jet Based 3D-Printing Technologies

The idea of “ink-jet” systems originated from computer-operated ink-jet printing, which recreates digital images by propelling ink droplets onto paper. This was adapted for pharmaceutical application by the replacement of the ink with liquid solutions containing APIs and excipients, and normal paper with edible sheets known as substrates. In this case, the major challenge, often underestimated, is the formulation of an API-containing ink with appropriate properties. Specifically, during a generic inkjet-based 3DP process, the ink must be sprayed at a set speed, and through specific motions, into droplets with precise sizes. Operative conditions must be well established to facilitate reliable jetting and homogeneous droplet formation with minimal satellites [88,92]. In addition, the choice of the solvent for the ink formulation, as well as the ink drying rate, could influence the solid state of the loaded API after deposition, and hence its bioavailability [93].

As illustrated in Figure 7, the ink-jet based 3DP technologies can be divided into two types: continuous (CIJ) and drop-on-demand (DoD) inkjet printing [56]. In the first case, the ink is sprayed, mainly through piezoelectric crystals, in a continuous flow. On the contrary, during a DoD process, the ink flow, either provided by a thermal or a piezoelectric device, is provided only as needed. This latter process can be also defined as drop-on-drop deposition (DOD) if the drops are allowed to deposit on each other to form a bed, and drop-on-powder deposition (drop-on-solid, DOS) if the droplets are allowed to deposit on the powder bed; this approach is also known as the TERIFORM^®^ process.

##### Drop-on-Drop (DOD)

**Figure 8:**
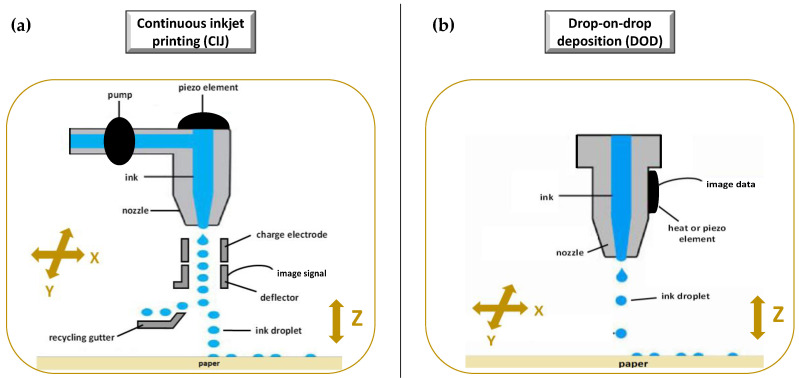
Ink-jet based printing technology (**a**) Continuous (CIJ), (**b**) drop-on-demand (DoD).

During a drop-on-drop deposition process, droplets of ink are sprayed from the thermal or piezoelectric print head, deposited on the thin layers, and then cured by cooling air or in the presence of high energy light (Figure 8b). In this case, to create support for overhang geometries, it is necessary to use additional material acting as support. The most common materials used for DOD are waxes and ceramics, and this low selection of materials certainly represents a disadvantage. By contrast, the main advantages of DOD are the instantaneous solidification, the efficacy, and the cost-effectiveness.

##### Drop-on-Solid (DOS)

**Figure 9:**
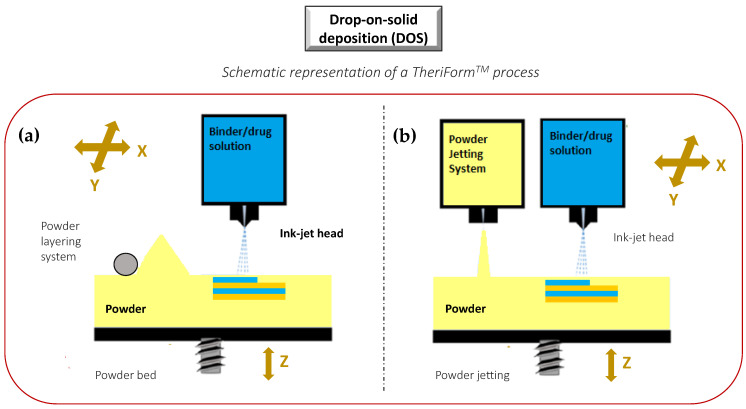
Illustration of a DOS deposition process with (**a**) powder bed layering system and (**b**) powder bed jetting system.

In a drop-on-solid process, droplets of ink sprayed from the print head bind the layer of the free excipient powder bed, while unbound powder particles act as a support material preventing the collapse of overhanging or porous structures. After each step, the formed object is lowered, and a layer of free powder is applied by a roller or powder jetting system and the process proceeds (Figure 9). The product quality attributes are strictly dependent on both ink and powder properties. The ink constituents, such as APIs, solvents, or excipients, can influence viscosity and droplet size, and thus the efficiency of powder binding. Instead, the particle size, flowability, and wettability of the powder bed, as well as the cohesion force between particles and printer components, mainly influence the layer height and, consequently, the final resolution of the printed object [56,94]. Moreover, printing speed, droplet volume, and distance from powder bed may play an important role, particularly in affecting the powder bonding between layers along the Z-axis, where they could negatively influence its mechanical strength [1].

The main advantage of such technique relies in the high similarity with wet granulation, presenting the possibility to use as starting materials various common excipients of solid dosage forms [95,96,97]. Similar binders work with a wide range of API powders allowing to greatly reduce the complexity of ink formulation. Besides the possibility of a precise location of an exact drug dose, this approach also allows an easy modification of the excipients within the powdered bed to obtain, in the same product, several compartments with different composition or mode of action. The main disadvantage of the DOS approach is represented by the need to perform different post-printing steps, such as drying to eliminate residual solvents and improve the physical resistance or unbound powder removal, and this latter step requires a specialized powder facility [1].

In the literature, there are a lot of examples concerning the application of the DOS method for the fabrication of tablets [97,98,99,100,101], and the greatest success of this technology was achieved in 2015 with just a tablet, namely Spritam^®^, based on ZipDose^®^ technology [4,5]. In contrast with conventional compression, the technology yields a product layer-by-layer without using compression forces, punches, or dies. During the process, a powder blend is first deposited as a single layer. Then, an aqueous binding fluid is applied, and interactions between the powder and liquid bind the materials together. The process is repeated several times to produce solid, yet highly porous, friable formulations, even at high dose loading (up to 1000 mg).

The DOS method could also be exploited in the large-scale production of modified release multicomponent tablets. In this case, small variations in porosity resulting from the different adhesion between the layers could alter the structural density of the produced tablets and, consequently, the drug dissolution profile and bioavailability. The technical solution could be the application of a greater amount of binder, which, however, causes an increase of drying time as well as the risk of limited removal of residual solvent [1]. This aspect strongly limits the application of DOS for the production of multicomponent modified release tablets with high quality.

#### 3.2.2. Laser Based 3D-Printing Technologies

The second set of 3DP technologies is that laser-based 3DP technologies. This group includes selective laser sintering (SLS), or selective laser melting (SLM), whose constructive assumptions are similar to the DOS method, and stereolithography (SLA), for which the object is built by the solidification of photosensitive liquids.

##### Selective Laser Sintering (SLS)

**Figure 10:**
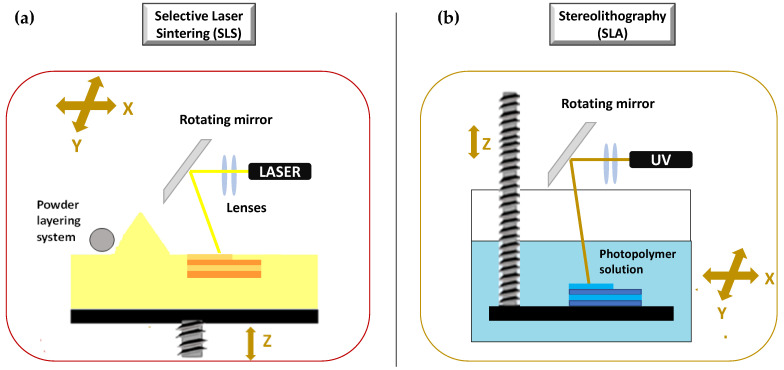
Illustration of SLS (**a**) and SLA (**b**) processes.

SLS is a laser based 3DP technique based on powder solidification by applying a high-energy beam [94,102,103]. As illustrated in Figure 10a, a layer of free powder is applied by roller and each layer is formed by sintering via laser beam that is able to heat just below melting temperature [1]. This technique can be applied to ceramic powders as well as to thermoplastic or metal powders. In this latter case, laser beam must melt the powdered bed and the specific technique is referred to as selective laser melting (SLM) [103].

Many are the advantages and the disadvantages of such a technique. Ideally, almost any dosage form can be fabricated by SLS with a high level of precision, accuracy, and resolution. In fact, even objects of several cubic centimeters (and hence rather large) can be built with a resolution down to 0.2 micron [104]. SLS can be successfully applied to produce porous, rapidly disintegrating, as well as modified release dosage forms without binding agent, with high drug loading efficiency and good mechanical properties. The latter aspect is very important, because it cannot be reached with other powder solidification methods such as DOS [1]. However post-printing processing is required as the object is built into a powder, and such a step requires specific powder removal procedures and facilities. Other disadvantages are due to the risk of API decomposition after exposure to laser beam, the high variability of mechanical properties, and the limited speed for sintering [1]. The drug degradation in particular has severely limited the use of SLS in the production drug-loaded devices, and a few examples are available [105,106,107,108,109,110,111]. Nevertheless, SLS has been used to process soft materials both in the bioprinting for tissue engineering and in the food industry [112].

##### Stereolithography (SLA)

The production of a 3D object by SLA is based on the controlled solidification of subsequent layers of resin by photo-polymerization via ultraviolet laser beam or light from a projector (digital light projector, DLP) [113]. During the SLA process, the printed object is bound to the built platform that is immersed in the photopolymer solution (Figure 10b). A digital mirroring device starts a chemical reaction in the photopolymer, which causes the cross-linking of the exposed area. The layer is traced on the surface of the resin.

As with SLS, SLA also is a highly versatile technique allowing to produce objects with high level of precision, accuracy, and resolution. The major disadvantage is represented by the need for post printing treatments to remove residual solvents, eventual supports, and in general, to improve final product properties, e.g., mechanical integrity. Another disadvantage is the potential health hazard due to the use of photo-sensible resins usually considered as carcinogens as well as responsible for a decrease of final product stability and its mechanical properties over time [56]. In addition, the systems exploiting this 3DP method require costly equipment and a long printing time. All these aspects have limited the application of SLA in the pharmaceutical and biomedical fields [114]. However, some applicative examples of SLA are described in the scientific literature [40,59,115,116], also in combination with other 3DP techniques [41,95,117,118].

#### 3.2.3. Nozzle Based 3D-Printing Technologies

The third group of 3D-printing technologies is represented by nozzle-based deposition systems allowing direct writing through extrusion. Such systems deposit ink direct through a nozzle to create a 3D pattern layer-by-layer with controlled composition and architecture [119]. They can be basically divided into processes based on material melting, such as fused filament fabrication (FFF), also referred to as fused deposition modelling (FDM), and processes without material melting, such as semi solid extrusion (SSE), also known as pressure-assisted microsyringe (PAM). Nozzle based 3DP technologies have been highly investigated due to their great versatility, reproducibility, and high scalability potential. A lot of papers in the literature have focused on the application of such techniques to develop pharmaceutical as well as biomedical products.

##### Fused Filament Fabrication (FFF)

**Figure 11:**
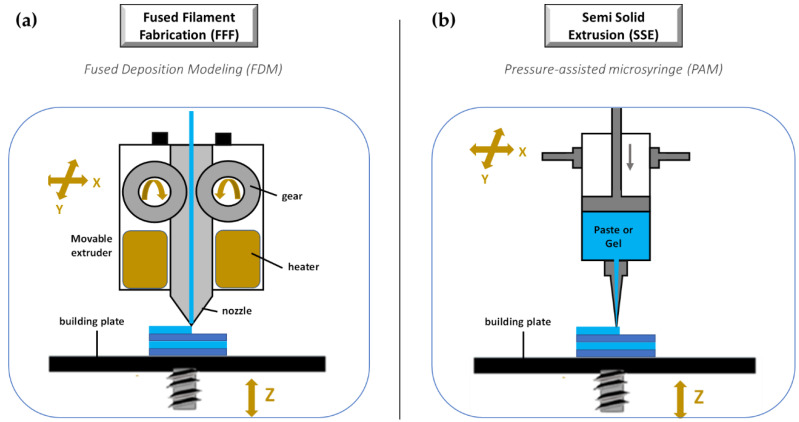
Illustration of FFF (**a**) and SSE (**b**) processes.

FFF is a really very investigated 3DP technique because it is cheap, easy to use, and readily available [119,120,121]. Its increasing popularity is mainly due to the progressive availability of compact sized and relatively inexpensive equipment [1,56]. During a FFF process, a thermoplastic polymeric material (mainly in form of filament) is extruded through a warmed-up nozzle and printed layer-by-layer (Figure 11a). Nozzle diameter varies from 0.2 to 0.4 mm, and it has an impact on the final resolution of 3D printed product. Generally, the width of the printed path corresponds to the nozzle diameter, while its height is equal to the half of the width. However, properties of the selected starting material as well as printer settings may induce modifications. During the process, the paths are arranged in layers until the formation of the final object, the resolution of which depends on layer height. Differently, the mechanical characteristics of the printed product are related to a number of outlines that build the external wall of the object and infill pattern (e.g., linear, or hexagonal).

The development of dosage forms and medical devices by the FFF approach requires a deep understanding of the printing process parameters as well as a thorough formulation study to properly select raw materials. Several critical material requirements need to be considered for their influence on FFF processability as well as 3D printed product quality. In more detail, filament mechanical properties (e.g., elastic modulus and strain at yield) and viscosity at the melted state mainly influence the extrusion step. Rheological properties, and particularly viscosity, surface tension, and relaxation dynamics, have impact above all on layer and intralayer adhesion, and thus on object precision and resolution. Finally, thermal properties (e.g., conductivity, heat capacity, coefficient of thermal expansion, and crystallinity), besides specifically driving process parameter set-up, are often responsible for fiber shrinkage and warpage [122]. Therefore, the careful evaluation of such aspects may avoid processing issues [1]. In general, the main disadvantages of FFF rely in the poor choice of starting materials which, as introduced, is limited to thermoplastic polymers, and the need of preparing filaments in advance, eventually loaded with the drug. Moreover, due to the elevated temperatures associated with this process, the potential risk of drug degradation is a significant issue hindering its use in pharmaceutical field.

##### Semi Solid Extrusion (SSE)

Differently from FFF, the SSE process involves a semisolid starting material (in the form of gel or paste) that is extruded through an orifice by compressed air pressure, a syringe plunger, or screw, depending on the specific equipment used, and deposited layer by layer (Figure 11b). Semisolid materials can be easily obtained by excipients commonly employed in the pharmaceutical industry by mixing them in optimal ratios with appropriate solvent(s) to obtain a viscosity suitable for printing. SSE does not require high temperatures but, using materials in form of pastes or gels, a further drying process is needed, implying shrinking or deformation of the printed product. The fabricated object may also collapse during 3D printing if a constructed layer did not harden sufficiently to withstand confinement of the successive layer. The technique is usually confined to a low resolution since an orifice with a size of 0.4–0.8 mm is typically employed. However, an accurate parameterization of the dispensing of the semisolid mass, as well as the use of nozzles smaller in diameter, allows to obtain dosage forms with a good resolution and mass uniformity [123,124]. The main advantage of SSE resides in the possibility to fabricate dosage forms with high drug loading. By using multi-syringe printing, “polypills” may also be obtained containing 3–5 APIs released with different kinetics [125,126].

The data shown earlier indicate that each 3DP approach presents specific advantages and disadvantages, and suggest the choice that must be made based on the properties of the starting materials as well as the drug to load and the desired performances for the final 3DP products, without forgetting system cost-effectiveness and realizable scale-up.

To give the reader a rapid comparison and insight into the different available techniques, the above discussed topics are summarized in Table 1.

## 4. Pharmaceutical and Medical Applications of Nozzle Based 3DP Techniques

### 4.1. FFF: Applications, Challenges and Perspectives

An overview of the main applications, common issues, challenges, progresses, and perspectives in FFF is presented in this section. In the last years, the application of FFF in manufacturing personalized drug dosage forms and medical devices has grown notably, and the number of analyzable research exploiting FFF is unbelievably high in the current scientific literature. A picture of the overall theme and a guideline for scientists working in this specific field is presented in Table 2, summarizing an accurate selection of both biomedical and pharmaceutical products realized via FFF-3DP technology. For each analyzed product, the main personalization possibilities (e.g., in terms of drug combination, dose, and release) as well as the encountered drawbacks (mainly related to the intrinsic limits of the FFF approach, such as high process temperatures) are highlighted.

Some case-studios reported in Table 2 (as considered more relevant) are examples of a successful response to different pharmaceutical needs and are deeply discussed below.

Generally, FFF printing requires a thermoplastic polymeric filament, the production of which is without doubt one of the most critical steps of the whole process, and responsible for a generally high production cost [127]. The filament for the 3D printing process must have key quality attributes, such as constant dimension, elasticity, and mechanical resistance [128]. During printing, filaments are bent and compressed between feeding and driving equipment. Therefore, filaments too brittle can be broken by the gears, whereas those too soft can be squeezed aside by the feeding gear [1]. There are a lot of ready-to-use filaments commercially available for FFF-3D printers. Their dimensions usually range between 1.75 mm and 2.85–3 mm and the most employed materials for standard filament production are thermoplastic polymers, such as acrylonitrile butadiene styrene (ABS), poly (lactic acid) (PLA), poly (vinyl alcohol) (PVA), polycaprolactone (PCL), high impact polystyrene (HIPS), polyethylene terephthalate glycol-modified (PET-G), polyurethanes (PU), and nylon (N). However, high quality filaments, produced from medical grade polymers as raw materials for research or industry are still scarce on the market, and those containing APIs are not available. This issue has led to the practice of offering consulting and/or manufacturing services for the development of drug loaded polymeric filaments from companies as well as academic research groups. Technically, various drug loading strategies are useful. However, each method may present limitations and should be chosen based on drug physico-chemical properties, printing material, and final desired performances of the printed product [75]. Drugs can be loaded into the preformed filament by soaking/swelling in a volatile solvent solution containing API, and subsequent drying [1,13,129,130,131,132]. The critical point of this method is to properly select the solvent or the solvent mixture that must be able to swell the polymeric material and at once solubilize the drug, allowing its loading within filament by diffusion [133]. At the same time, this approach is simple and easy to realize, but generally limited to the preparation of filaments with low drug content [1].

**Figure 12:**
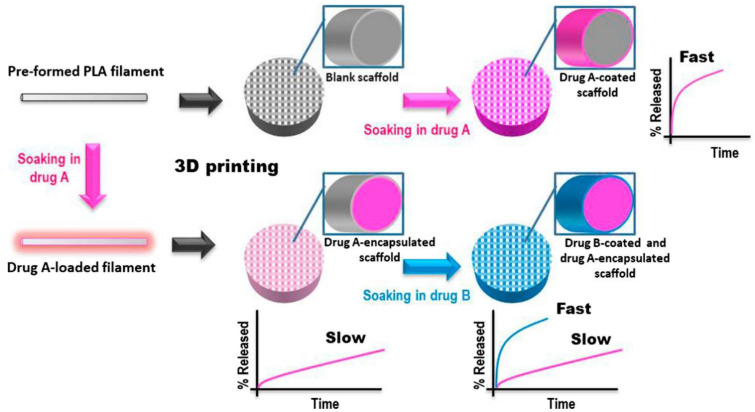
Drug loading by soaking of either preformed PLA filaments or 3D printed scaffolds. Reprinted with permission from reference [134]; copyright (2019) Elsevier B.V.

A challenge of regenerative medicine is to realize controlled drug-releasing tissue engineered platforms able to exert both an initial burst release as well as a sustained or long-term release of the loaded drug/s. In this case, a sequential or multiphasic release patterns is required, which may aid to enhance the speed, quantity, and quality of tissue regeneration. With this in mind, recently, Farto-Vaamonde et al. explored the soaking/swelling of PLA to personalize the steroidal anti-inflammatory drugs (SAIDs) release profile from scaffolds intended for regenerative purposes [134]. Two different drug loading strategies were exploited (see Figure 12). In the first case, to obtain feedstock for 3D FDM printer, the soaking of PLA filaments into a volatile solvent mixture containing the drug followed by drying was applied. The second strategy consisted in first printing the 3D PLA scaffolds followed by soaking in a suitable SAID solution. Results showed that during 3DP of drug loaded PLA filament, the melting of PLA contributes to the efficient encapsulation of the SAID inside the printed strand, leading to a sustained drug release profile from the scaffold for several months. Differently, 3D PLA scaffolds, loaded after printing with either prednisolone or dexamethasone, show that the coating of the scaffold strands with a layer of crystalline drug nanoparticles, consequently leading to a burst rapid release. This evidence led us to design a dually loaded scaffold exhibiting distinct drug release patterns. Dexamethasone loaded within the strand core showed a sustained release, whereas prednisolone loaded on the strand surface gave an immediate release thanks to the combination of two loading methods.

As reported in Table 2, many other examples of thermoplastic polymeric filaments loaded with drug/s via soaking/swelling and involved in the manufacturing of different dosage forms, as well as medical devices, can be found in the literature.

To produce filaments with higher drug content and mechanical properties suitable for 3DP, commercially available filaments can be shredded or milled with APIs by hot melt extrusion (HME). In this case, different additives, such as plasticizers, fillers, and lubricants can also be added to API-polymer blend to improve both the processability and the final printability of the extruded filaments [135]. Clearly, drug loading within the extruded filaments depends on physicochemical properties of drugs and excipients as well as the extruder construction (i.e., single-screw or twin-screw extruder, screw segment arrangement and size, types of barrel surfaces, types of thermocouple junctions). Moreover, process parameters (i.e., extrusion temperature, screw speed, torque, feed rate) [1,136] may affect drug charging.

Table 2 summarizes various materials exploited by HME technology to produce drug loaded filaments. Good performances have been shown by PCL [137], PLA [138], thermoplastic polyurethane (TPU) [139], poly (ethylene vinyl acetate) (EVA) [140], polyethylene oxide (PEO) [141], Eudragit^®^ L100, Eudragit^®^ RL, Eudragit^®^ PO [142], Soluplus^®^ [143,144], HPC [145], hydroxypropyl methylcellulose (HPMC) [146], ethyl cellulose (EC) [143], used alone or in combination [121,147]. However, Poly (vinyl alcohol) (PVA) remains the most investigated. PVA is a water-soluble semi-crystalline polymer prepared by the partial or complete hydrolysis of the acetate group from polyvinyl acetate. PVA is a safe excipient highly employed in pharmaceutical technology to develop drug delivery systems, particularly tablets [148,149,150]. PVA is largely used as solubilizer, base, binder, coating agent, sugar coating, adhesive, thickener, etc. Various biomedical applications are known, e.g., to realize contact lenses, synthetic tear eye-drops, or surgical sponges [151,152,153]. Recently, Wei et al. [133] proposed PVA to produce tablets with a rapid release profile of carvedilol and haloperidol (both weakly basic and poorly water-soluble) via hot melt-extruded filaments and FFF printing. This study discusses in depth the main development issues of using PVA, namely the high processing temperature and limited drug-polymer miscibility. The interest of this research resides in the testing of the miscibility of selected drugs with PVA, with and without added sorbitol as plasticizer. The aim was to verify whether any amorphous solid dispersion (ASD) was formed able to promote a rapid and pH-independent dissolution. Results showed a miscibility of carvedilol and haloperidol with PVA of, respectively, ~20% and <10%. Specifically, PVA provided complete drug release from 3D printed tablets with 10% and 20% carvedilol and 60% infill in ~45 min at both pH 2 and 6.8.

Another interesting example of systematic testing of PVA by HME technology was the development of PVA filaments loaded with ciprofloxacin hydrochloride in different concentrations to use as feedstock for a 3D FDM printer and to produce a printlet (3D-printed-tablet) [154]. With this aim, several solid mixtures were prepared, using five PVA batches (4000–5000 µm, 1000–2000 µm, 600–1000 µm, 250–600 µm, <250 µm) and ciprofloxacin hydrochloride in different ratios. This study on the influence of polymer size distribution on drug loading capability showed that the best results in terms of HME processability are obtained for the finest PVA batches. Particularly, moderately fine particles (250–600 µm) showed a positive impact on mixing–extrusion–printing steps, allowing a complete adhesion of the drug on the polymer surface and a greater drug homogeneity of both filaments and printlets.

**Figure 13:**
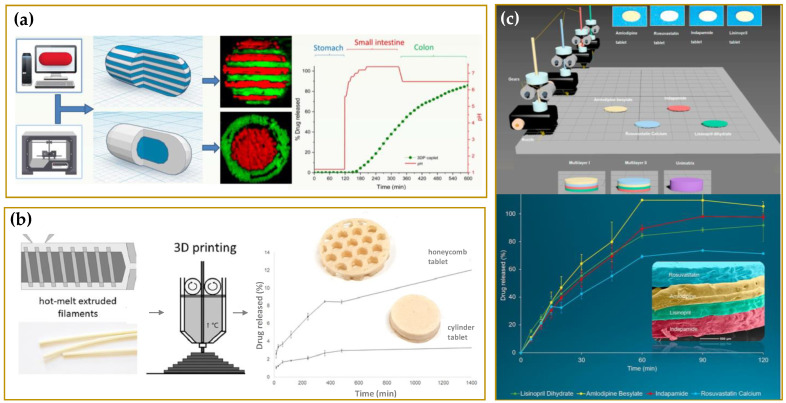
Examples of 3D printed oral dosage forms produced by FFF technique. (**a**) Multilayer device and Duo-caplet. Adapted with permission from reference [155]; copyright (2015) American Chemical Society; (**b**) HME polycaprolactone-based filaments intended for 3D-printing of tablets with a lattice (“honeycomb”) structure. Reprinted with permission from reference [136]; copyright (2020) Elsevier B.V.; (**c**) Cardiovascular ‘Polypill’ loaded with four different drugs. Reprinted with permission from reference [159]; copyright (2018) Elsevier B.V.

In addition Goyanes et al. explored such an approach in different researches. For example, this research group successfully obtained through HME paracetamol (4.3 and 8.2%) or caffeine (4.7 and 9.5%) loaded filaments of PVA with characteristics suitable for FFF. The produced drug loaded filaments were used as feed for a multi-nozzle FFF 3D printer to produce oral drug delivery devices with different inner structures (multilayer device and DuoCaplet, see Figure 13a). The obtained devices exhibited unique drug release profiles, which would be challenging to obtain by a conventional manufacturing method [155]. In another work, Goyanes et al. employed this strategy to produce a system intended for the treatment of inflammatory bowel disease. 3D-printable PVA filaments loaded with budesonide were engineered into caplets (capsule-shaped tablets) containing 9 mg budesonide using a FDM 3D printer. The caplets were subsequently overcoated with a layer of enteric polymer (Eudragit L100) [156]. Moreover, Goyanes et al. [157] prepared filaments based on hypromellose acetate succinate (HPMCAS) incorporating up to 50% of paracetamol, thanks to the addition to the polymeric matrix of methylparaben as plasticizer and magnesium stearate as lubricant. The filaments were obtained using a single-screw extruder and were subsequently processed by FDM in enteric printlets, exhibiting delayed drug release profiles in biorelevant bicarbonate dissolution media. Interestingly, the specific drug release performance from each group of 3D printed tablets was dependent on the polymer composition (HPMCAS grade, that is LG, MG or HG), drug loading (5% or 50%), and the internal structure of the formulations (infill percentage of 20% or 100%).

Zang et al. [147] successfully developed cellulose-based solid-dispersion filaments with the API (Acetaminophen) dissolved or dispersed in the polymer matrix by a twin-screw extruder. Filaments were obtained starting from binary polymer blends of HPMC E5 and EC N14 with either HPC EF and LF, Soluplus^®^, or Eudragit^®^ L100. All the extruded filaments showed good mechanical properties and they were printed well via FFF to produce controlled-release tablets.

Further, thermoplastic polyurethanes (TPU) [158] have potential good performance in HME. Melt-extruded filaments based on different grades of hydrophilic and hydrophobic TPU may load a high quantity of the crystalline drugs, such as theophylline and metformin (up to 60%). The high content of the crystalline drug resulted in a very rough surface of the produced filaments. However, after API milling, filaments showed a smoother surface as well as consistent diameter, good mechanical properties, and were therefore successfully converted via FFF into tablets whose drug release performances proved to be mainly influenced by matrix composition and infill degree.

In the literature, there are also many reports on the use of PCL for the development of HME filaments intended for a pharmaceutical FFF-3D printing. PCL is a biodegradable, water-insoluble, FDA-approved polymer with a low melting point (T_mp_ 50–60 °C) [160] and highly employed as a carrier to produce patient-specific, 3D-printed, drug-eluting implants (e.g., scaffolds [161,162], gynecological devices [163,164], antimicrobial wound dressings [165], and biodegradable/bioabsorbable stents [166]. More recently some researchers have investigated the potential use of such polymer in combination with natural gums (e.g., arabic gum, ARA) to prepare hot-melt extruded polycaprolactone-based filaments intended for 3D-printing of tablets. Particularly, Viidik et al. [136] used a single-screw hot-melt extruder to produce drug loaded filaments of PCL and ARA, and, in particular, with 20%, 30%, and 40% (*w*/*w*) of indomethacin (IDM) and theophylline (THEO). These HME filaments exhibited properties suitable for FFF, such as smooth surface and sufficient mechanical properties. Special attention was paid to the uniform distribution of the drug and the overall homogeneity of the HME filaments as well as to their physical stability. Results showed that, if properly stored, the extruded filaments can be successfully 3D-printed into tablets. Furthermore, by modifying the size, shape, and texture of the cylinder-shape 3D-printed tablet (i.e., a lattice “honeycomb” tablet form, see Figure 13b), an aspect easily approachable by means of 3D printing, the release of the loaded drug can be significantly improved.

**Figure 14:**
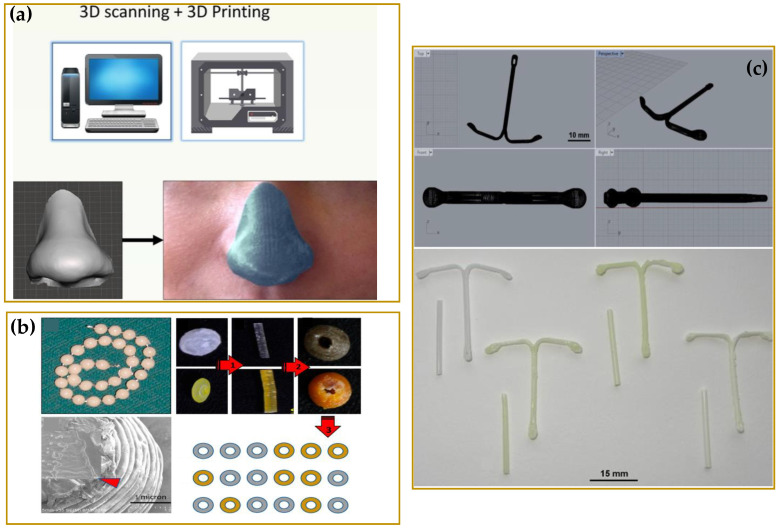
Examples of 3D printed medical devices produced by FFF technique. (**a**) Antimicrobial polycaprolactone wound dressings. Reprinted with permission from reference [165]; copyright (2017) Elsevier B.V; (**b**) Antibiotic loaded implants. Reprinted from reference [167]; copyright (2017). (**c**) Long-lasting implantable drug loaded intrauterine system. Reprinted with permission from reference [164]; copyright (2016) Elsevier B.V.

The factors affecting the uniform drug distribution within PCL after HME and FFF 3D-printing processes was deeply investigated by Holländer et al. [164]. In this study, indomethacin-loaded, PCL-based filaments with three different drug contents, namely 5%, 15%, and 30%, were produced by hot-melt extrusion. The extruded filaments were further used to 3D print long-lasting implantable drug loaded IUS (intrauterine system, see Figure 14c). Results showed that the amount of the loaded drug affected the filament roughness, solid state, printability, and drug release performances of the 3D printed devices. The higher the drug content, the higher the crystallinity degree, and the lower the percentage of drug dissolved in the polymer, and hence released.

To ensure a greater uniformity of drug loading within the extruded filament, an alternative way to the direct extrusion of the physical mixture API/polymers (and additives) could be the loading of the drug in solution. In this case, one of the most challenging process steps is the selection of a solvent or a solvent mixture able to dissolve both the API and polymer/s. An interesting application is the possibility to produce antimicrobic dressing. For example, Muwaffak et al. [165] explored such a strategy to load antimicrobial metals, such as zinc, copper, and silver, inside PCL, and produce polymeric filaments for 3DP. In this work, HME technology using a Filabot filament hot-melt extruder with a single screw and a 1.75 mm nozzle head was used to extrude pellets obtained by vacuum-drying solutions containing PCL and Silver (10% loading *w*/*w*), Copper (10% and 25% loading *w*/*w*), or Zinc (10% and 25% loading *w*/*w*). Tetrahydrofuran (THF) and dichloromethane (DCM) were selected as solvents to dissolve the PCL pellets, which were added to the metal solutions (aqueous, methanolic, and ethanolic for silver, copper, and zinc, respectively) and obtain homogeneous mixtures. The extruded filaments were then processed by an FDM-3D printer to manufacture wound dressings with different shapes, whose digital models were obtained by 3D scanning. Release data determined by inductively coupled plasma atomic emission spectroscopy showed for all the different metal dressings a fast release (up to 24 h) followed by slow release (up to 72 h), while the best bactericidal properties were found for the dressings based on silver and copper.

HME-FFF have great potential. However, the filament production step plays a critical role in consuming time and materials. So, in the last years, the possibility to print in 3D by FFF, avoiding the intermediate HME process necessary for the development of filaments, has been investigated. Recent advances in this field have made available equipment able to directly 3D print by FFF technology starting materials in the form of powders or pellets [56,168,169]. Goyanes et al. reported for the first time in 2019 a novel single-step printing process to produce 3D printed tablets directly from powdered materials, and specifically hydroxypropylcellulose and itraconazole. All the obtained printlets showed good mechanical and physical characteristics and no drug degradation. This possibility may represent a notable advantage from a productive point of view. Indeed, it could revolutionize the preparation of amorphous solid dispersions (ASD) as final formulations and it may be especially suited for preclinical studies, where the amount of drug is often limited [170]. Some machines based on this single step approach are already on the market. CellInk^®^, for example, has developed a thermoplastic printhead for its BIO X^TM^ 3D-printer for whose use the cartridge must be loaded directly with a powder. To guarantee a uniform product printing when starting from thermoplastic materials in mixture among them or with APIs, many additional aspects must be considered, but all the profuse efforts being made may help in the near future to maximize the already high versatility of the FFF 3DP technique.

**Table 2:** Literature examples on dosage forms and medical devices produced via FFF 3DP. In evidence for each product—performances; —starting materials (polymers/excipients and APIs); —drug loading strategy; —challenges and drawbacks.

3D Printed Product	Properties and Performances	API/s	Excipients	Filament Production Technique	Challenges and Drawbacks	Ref.
Polypills	○ Cardiovascular polypill containing three different drugs, each with an optimized release profile	○ Lisinopril dihydrate (LD), Indapamide (I), rosuvastatin calcium (RC), and amlodipine besylate (AB)	○ PVA Plasticized with sorbitol (without water, 170 °C) and with water and sorbitol (90 °C) Titanium dioxide (TiO_2_) was added to the formulation of all individual drug filaments except amlodipine besylate, as it catalyzed its chemical degradation	○ HME (twin-screw extruder)	○ To avoid drug degradation The use of a higher processing temperature had a negative effect on the integrity of the model drugs particularly for lisinopril ○ To find the optimal plasticizer The authors explored an evaporable plasticizer, that can initially facilitate polymer extrusion, and then followed by its partial or complete removal (in a secondary step) is able to restore filament mechanical rigidity towards printer ready robustness.	[159]
Tablets	○ Tablets with controlled release profile	○ Isoniazid (INZ)	○ HPMC, HPC, Eudragit^®^ RS PO, RL PO and L Triethyl citrate (TEC), Kolliphor^®^ TPGS (vitamin E polyethylene glycol succinate, d-alpha tocopherol) were used as plasticizers in some formulations	○ HME (twin-screw extruder)	○ Filament hygroscopicity and fragility, which negatively affect printing process	[171]
	○ Tablet with controlled release profile Dissolution profiles can be modified varying infill percentage	○ Fluorescein (F)	○ PVA	○ API loading in pre-formed filaments by soaking A: soaking in EtOH solution of F (2% *w*/*w*) for 24 h; B: drying in oven (60 °C) for 1.5 h and storage in a vacuum desiccator until printing	○ Very low API loading in the strands Final tablet drug content = 0.29%	[132]
	○ Tablet with controlled release profile	○ Budesonide (B)	○ PVA (cut, milled and extruded) Eudragit L100 (as coating applied with fluid bed-coating)	○ HME (single-screw filament extruder)	○ Low drug loading Probably due to the adherence of drug to the walls of the container on transfer to the hopper of the HME and the walls of the barrel during extrusion, and to irregular extrusion of components (single-screw extruder).	[156]
	○ Multiple drug containing tablets Multilayer device DuoCaplet	○ Acetaminophen (A) and Caffeine (CAFF)	○ PVA	○ HME (single-screw filament extruder)	○ The adhesion of fine drug powder to the equipment It’s important to have comparable particle size	[155]
	○ Tablets	○ Acetaminophen (A) and Caffeine (CAFF)	○ PVA	○ HME (single-screw filament extruder)	○ The adhesion of fine drug powder to the equipment	[172]
	○ Tablets with controlled release profiles	○ 4-Aminosalicylic acid (4-ASA) and 5-Aminosalicylic acid (5-ASA)	○ PVA	○ API loading in pre-formed filaments by soaking.	○ Very low API loading in the strands	[13]
	○ Tablets with delayed release profiles	○ Prednisolone (PRED)	○ PVA	○ API loading in pre-formed filaments by soaking. a_soaking in MetOH solution of PRED for 24 h; b_drying The yielded PRED loaded filament showed a drug loading of approximately 1.9% *w*/*w*.	○ Low filament drug loading necessity to modify volume of 3D printed tablet to obtain target tablets’ doses (2,3,4,5,7.5,10 mg)	[131]
	○ Bilayer tablet with dual controlled drug release for tuberculosis treatment	○ Isoniazid (INZ) and rifampicin (RFC)	○ HPC and HPMCAS (PEG was added to the RFC/HPMCAS filament, as a plasticizer to enable extrusion at lower temperature and thus minimize degradation of RFC)	○ HME (twin-screw extruder)	○ Small reduction of drug amounts in the extruded probably due to the stickiness of the drugs in the extruder barrels—For an optimal drug release control, infill density, and covering layers must be properly selected	[173]
	○ Tablets EE_Immediate release HPC_Immediate releas ERL_extended release ERS_extended release ERL + ERS_ext. release	○ Theophylline (THEO)	○ Eudragit E (EE), HPC SSL (HPC), Eudragit RL (ERL), Eudragit RS (ERS), TEC	○ HME (twin-screw extruder)	○ Nozzle Clogging To control T during HME process for avoiding clogging, initial T is higher than the extruding T	[174]
	○ Bilayer oral solid dosage form	○ Metformin (MET) and glimepiride (GLP)	○ Eudragit^®^ RL PO and PVA PEG 400, TEC, and citric acid monohydrate were added as plasticizers to Eudragit^®^ RL PO whereas PLA was added in some formulations to improve mechanical strength of the filament. Mannitol (MANN) was used as plasticizer for PVA; in addition, calcium stearate was added to prevent excessive die swell and facilitate extrusion	○ HME (single-screw extruder for all the tested formulations and twin-screw extruder for the optimized ones)	○ To find the appropriate combination of filament hardness and elastic modulus	[175]
	○ Immediate release tablets	○ Theophylline (THEO) and dipyridamole (DPR)	○ PVP (TEC as plasticizer agent; talc as thermostable filler)	○ HME (twin-screw extruder)	○ Partial PVP degradation at the recommended 3D printing temperatures; Difficulties to obtain by HME stable structure due to poor flow of the polymer from the hot nozzle of the printer and the formation of collapsed structure To obtain by HME stable structure and allow rapid solidification of the filament from the hot nozzle, talc was added a thermostable filler to the composition of the filament.	[176]
	○ Abuse Deterrent Immediate Release Egg-Shaped Tablet (Egglets)	○ Metformin hydrochloride (MET HCl)	○ PVA, Klucel™ (HPC), Kollidon^®^VA64 (copovidone), Affinisol™15LV, and Kollicoat^®^ IR with/without plasticizer Sorbitol was used as plasticizer	○ HME (twin screw extruder)	○ To obtain by HME easily printable filaments (with adequate mechanical strength, flexibility, and elasticity); To 3D print tablets hard enough to resist to any physical manipulation applied by using common household equipment	[177]
	○ Shell-Core Delayed Release Tablets	○ Theophylline (THEO) Budesonide (B) Diclofenac (DCF)	○ PVP and EudragitL100–55 (EL) Core 1 (THEO) PVP + TEC + TALC Core 2 (B) PVP + TEC + TALC Core 3 (DCF) PVP + TEC + TALC Enteric Shell EL + TEC + TALC	○ HME (twin-screw extruder)	○ Frequent block of PVP fil. (core) due to the sticking of the fil. to the internal wall of the nozzle. To overcome this problem several additives with high boiling point (castor oil, oleic acid or PEG 400) were incorporated in PVP filament composition. Castor oil was chosen as lubricant	[135]
	○ Immediate release tablet	○ 4-ASA 5-ASA Captopril (CPT) Theophylline (THEO) Prednisolone (PRED)	○ Eudragit EPO, TEC + TCP TCP, tribasic calcium phosphate was used as thermostable filler)	○ HME (twin-screw extruder)	○ The ability to adapt polymer with low Tg, such as EPO values to FDM 3D printing. To overcome this issue, a non-melting filler was added to methacrylic matrix.	[178]
	○ Extended drug release Tablets	○ Acetaminophen (A)	○ HPMC, HPC, Ethyl cellulose (EC), Soluplus (SLP), Eudragit L100 Optimized polymeric blends: HPMC + EC HPMC + HPC HPMC + SLP HPMC + EL100 EC + SLP HPC + EC PLA without drug deposition was used as the reference standard	○ HME (twin-screw extruder)	○ To obtain printable filaments, with high breaking stress, high stiffness, and long breaking distance	[147]
	○ Enteric Tablets	○ Acetaminophen (A)	○ HPMC AS HPMC + MP + MS HPMC + MP + MS MP_Methyl Paraben was used as plasticizer MS_Magnesium stearate was used as lubricant	○ HME (single-screw extruder)	○ Flexibility and resistance→only 15% and 5% *w*/*w* MP were found to provide good physical characteristics for all the HPMCAS grades tested.	[157]
	○ Disks based on different materials to test the possibility to use them as main components of different pharmaceutical products, for example to produce immediate (KIR or PEO), pulsatile (HPMC, HPC, PVA, SLP), enteric (HPMCAS, EL) or delayed (ERL, EC) release tablets	○ Acetaminophen (A), Furosemide (FS)	○ Kollicoat^®^ IR (KIR), Polyethylene oxide (PEO), HPMC, HPC, PVA, Soluplus (SLP), HPMCAS, Eudragit L (EL), Eudragit RL (ERL), Ethyl cellulose (EC) and various plasticizers	○ HME (twin-screw extruder)	○ Filament diameter calibration →twin screw extruder was equipped with a custom-made aluminum die Mechanical properties→problems of rapture or wrapping→feeding mechanism of the printer was modified by replacing the standard spring with an of lower stiffness and plasticizer amount was adjusted	[144]
	○ Swellable/erodible capsular device/shells device for oral pulsatile release of drugs	○ Acetaminophen (A)	○ HPC (and PEG 1500 as plasticizer)	○ HME (twin-screw extruderr	○ Filament diameter calibration Non-calibrated filaments led to the formation of air bubbles within the printed material or in clogging of the tip. So, the twin-screw extruder was equipped with a custom-made aluminum die and coupled with a purposely designed pulling/calibrating device	[179]
Pediatric-friendly printlets	○ Chewable tablets of different shapes (heart, ring, lion, bottle etc.) inspired by the Starmix gummy sweets (HARIBO plc.)	○ Indomethacin (IDM)	○ Hypromellose acetate succinate (HPMCAS) and polyethylene glycol (PEG)	○ HME (twin-screw extruderr	○ Filament quality and flexibility PEG was added as plasticizer to facilitate better extrusion processing and enhance the strand flexibility	[180]
Films	○ Mucoadhesive buccal films for unidirectional drug release	○ Diclofenac sodium (DCFS)	○ PVA (xylitol as plasticizer); Ethylcellulose (EC) (TEC as plasticizer) Chitosan (C) (as a permeation and mucoadhesion enhancer)	○ HME (Single-screw extruder)	○ The realization of a unidirectional drug release profile EC was added as hydrophobic printed layer and a backing layer (commercial wafer sheets) was also selected as efficient barrier to drug release from the corresponding surface	[181]
Medical devices, implants, etc.	○ Flexible personalised-shape anti-acne drug loaded devices Nose-shape mask	○ Salicylic Acid (SA)	○ NinjaFlex^®^ (NF), Flex EcoPLA™ (FPLA) and polycaprolactone (PCL)	○ HME (Single-screw extruder)	○ To obtain printable drug loaded filaments After HME process, filaments became red-brown and brittle Drug degradation at high temperatures (extrusion and 3DP)	[42]
	○ Antibiotic loaded implant devices (disks, beads, and pellets)	○ Gentamicin (GS)	○ Polylactic acid (PLA), halloysite nanotubes (HNTs)	○ HME (Single-screw extruder)	○ None in particular no problems experienced during the extrusion process. There was no clogging of the extruder or print heads and fabrication of HNT doped and drug doped HNTs into beads, disks and filaments occurred with high fidelity	[167]
	○ Bioactive and absorbable surgical screws, pins, and bone plates for localized drug delivery	○ Gentamicin (GS) and methotrexate (MTX)	○ PLA	○ HME (Single-screw extruder)	○ Flexural and compressive strength of the final products after drug loading/addition	[74]
	○ 3D-printed O, Y and M-shaped vaginal rings	○ Progesterone (PRG)	○ PLA/PCL PEG and Tween 80 were used as additives	○ HME (single-screw extruder)	○ To find the optimal mixtures of PLA/PCL for obtaining stiff enough filaments as well as with good thermoplastic properties and elasticity To enhance hydrophilicity of 3D printed rings Tween 80 was selected for its hydrophilic effect on PLA/PCL scaffolds To maintain physical properties of the extruded filaments loaded with the drug several cracks were observed on filament surface	[45]
	○ 3D-printed functional disks able to prevent to prevent biofilm formation	○ Nitrofurantoin (NTF)	○ PLA	○ HME (single-screw extruder)	○ To obtain drug loaded filaments with smooth surface	[138]
	○ 3D-printed antimicrobial nanocomposite disks	○ Silicon Dioxide (SiO_2_) nanoparticles (NPs)	○ PLA	○ HME (single-screw extruder)	○ To optimize nano silica content for obtaining a novel nanocomposite material printable via FFF, and exhibiting enhanced mechanical, morphological, thermal, and antibacterial properties compared to PLA alone	[182]
	○ Scaffolds for tissue regeneration applications	○ Prednisolone (PRED) and Dexamethasone (DEX)	○ PLA	○ API loading in pre-formed filaments/or printed scaffolds by soaking	○ To find the adequate solvent mixture able to dissolve the APIs and, at the same time, swell the polymer	[134]

Abbreviations. APIs: Lisinopril dihydrate (LD); Indapamide (I); Rosuvastatin calcium (RC); Amlodipine besylate (AB); Isoniazid (INZ); Fluorescein (F); Budesonide (B); Acetaminophen (A); Caffeine (CAFF); Salicylic Acid (SA); 4-Aminosalicylic acid (4-ASA); 5-Aminosalicylic acid (5-ASA); Prednisolone (PRED); Rifampicin (RFC); Theophylline (THEO); Metformin (MET); Metformin hydrochloride (MET HCl); Dipyridamole (DPR), Captopril (CPT); Glimepiride (GLP); Diclofenac (DCF); Diclofenac sodium (DCFS); Gentamicin (GS); Methotrexate (MTX); Progesterone (PRG); Nitrofurantoin (NTF); Dexamethasone (DEX). Excipients: Poly (lactic acid) (PLA); Polyvinyl alcohol (PVA); Hydroxypropylcellulose (HPC); Hydroxypropylmethylcellulose (HPMC); Hydroxyl propyl methyl cellulose acetate succinate (HPMCAS); Ethyl cellulose (EC); Poly (methyl methacrylate) derivatives, Eudragit^®^ (E); Triethyl citrate (TEC); Tribasic calcium phosphate (TCP); Polyethylene oxide (PEO); Polyvinyl caprolactam-polyvinyl acetate-polyethylene glycol graft copolymer, Soluplus^®^ (SLP); polyethylene glycol (PEG); polycaprolactone (PCL).

### 4.2. SSE: Applications, Challenges and Perspectives

Apart from FFF, EBP (extrusion-based 3D printing) comprises semisolid-extrusion technology (SSE). This section provides an overview of advanced design solutions, strengths and weaknesses, opportunities, and challenges in SSE printing.

The current state of art based on a detailed literature analysis regarding SSE capability to develop pharmaceutical and medical products is summarized in Table 3. Generally, SSE is based on the pressure-assisted extrusion of a paste or gel through a syringe-based printing head followed by deposition of the material on the printing platform. As it clearly emerges from Table 3, such a technique has been highly explored in the production of various pharmaceutical dosage forms, different types of tablets, and polypills (e.g., loaded with single API or multiple APIs, floating, orodispersible or chewable), many of which for pediatric requirements. Moreover, an interesting application is the manufacturing of orodispersible films [183].

**Figure 15:**
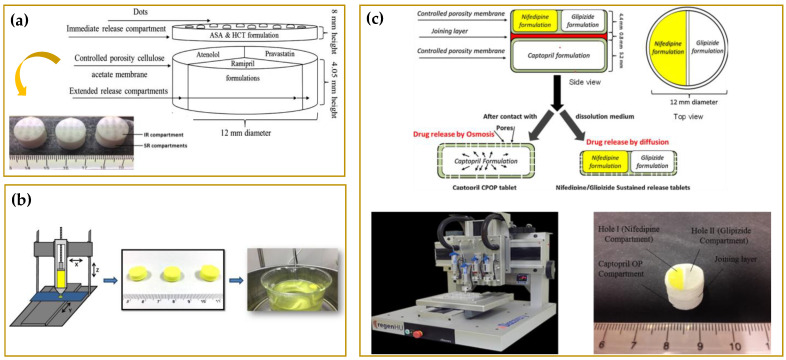
Examples of 3D printed oral dosage forms produced by SSE technique. (**a**) Polypill loaded with five different APIs. Reprinted with permission from reference [126]; copyright (2015) Elsevier B.V.; (**b**) Gastro-floating tablets of dipyridamole. Reprinted with permission from reference [184]; copyright (2017) Elsevier B.V; (**c**) Polypill designed as an osmotic pump containing captopril, and with other two compartments acting as sustained release platforms for nifedipine and glipizide. Reprinted with permission from reference [125] copyright (2015) Elsevier B.V.

A remarkable example of a five-in-one polypill with strictly controlled drug release has been designed by Khaled et al. [126], who developed a novel tablet with a complex geometry able to deliver five APIs with two independently well-defined release profiles. The first three compartments were designed to release in a sustained manner pravastatin, atenolol, and ramipril. These sections were covered with an immediate release compartment of aspirin and hydrochlorothiazide (see Figure 15a). The combination of such drugs in a single polypill for the prevention and treatment of cardiovascular diseases allows to optimize therapy as well as patient compliance while avoiding incompatibility issues thanks to the physical separation of each drug within the compartments. The three drugs, atenolol, pravastatin, and ramipril, mixed with a hydrophilic matrix (HPMC) were extruded into the segmented compartments, whose barrier for the APIs sustained release was obtained by the extrusion of a hydrophobic cellulose acetate membrane. Aspirin and hydrochlorothiazide, mixed with a disintegrant and other excipients, were extruded directly on the top of the cellulose acetate compartments to realize the immediate release compartment. In another work, Khaled et al. used SSE to design a polypill with compartments exhibiting release mechanisms of a different type, namely osmotic release through a controlled porosity shell and diffusion through gel layers [125]. As reported in Figure 15c, the captopril-loaded core of the polypill obtained using hypromellose hydro-alcoholic gel as binder and sodium chloride as osmogen acts as an osmotic pump, whereas the other two compartments realized with a HPMC hydrophilic matrix act as sustained release platforms of nifedipine and glipizide.

SSE has also been exploited for the fabrication of gastro-floating tablets to improve the bioavailability, and thus therapeutic efficacy of some drugs. Li et al. [184] successfully developed gastro-floating tablets with a fine lattice internal structure (see Figure 15b) loaded with dipyridamole, a poorly water-soluble drug with a short biological half-life. Tablets were prepared starting from pastes of hydroxypropyl methylcellulose (HPMC K4M) and hydroxypropyl methylcellulose (HPMC E15), two traditional pharmaceutical excipients, and applying three different infill percentages (30%, 50%, and 70%).

In the last few years, 3D printing via SSE has become consistently popular to produce oro dispersible tablets (ODT) [185]. Recently, some researchers explored this technique to produce carbamazepine (CBZ) loaded ODTs using cyclodextrins to increase the solubility and bioavailability of the drug [186]. Hydroalcoholic wet masses containing mixtures of CBZ, hydroxypropyl-β-cyclodextrin (HPβCD), and various cellulose ethers enabled in situ drug–HPβCD complex formation with suitable rheological properties for SSE-3DP. In more detail, the authors focused their attention on: (i) the choice of the best binder agent among HPMC E4M, HPMC F4M, sodium carboxymethylcellulose, and PVP K25; (ii) the optimal amount of wetting liquid; (iii) the influence of the croscarmellose sodium on both tablet disintegration time and mechanical resistance; and (iv) the printing operative parameters (e.g., tablet size, pore size, perimeters, total number of layers, infill pattern, flow speed, infill speed, and travel speed). The most homogeneous printable pastes were obtained using HPMC F4M as binder, water: ethanol 90:10 *v*/*v* as moistening liquid, and croscarmellose sodium 2.5% *w*/*w* as disintegrant. The porosity of the extruded tablets was properly regulated through slicing and printing parameter set-up, allowing to obtain porous tablets with fast release characteristics as well as physical properties appropriate for handling. This research highlights the close relationship between the paste rheology properties and its performance as 3D ink for SSE, in terms of consistency and homogeneity of the strand and printing resolution, and how much rheological analysis of the wet masses is a pre-condition to identify the best printable compositions.

SSE is an important tool in personalizing pediatric formulations. Indeed, the number of papers reporting the use of SSE 3DP in the manufacturing of pediatric-friendly chewable printlets for the oral administration of both hydrophilic and lipophilic drugs is growing. Goyanes et al., for example, evaluated for the first time the use of SSE 3D printing in a hospital setting for the administration of isoleucine in the form of personalized chewable printlets to treat a rare metabolic disorder, maple syrup urine disease (MSUD), in pediatric patients [187]. Isoleucine blood levels after six months of treatment with personalized chewable formulations prepared at the hospital by automated 3D printing were comparable to those obtained with conventional capsules prepared by manual compounding. However, the 3D printlet therapy caused mean levels closer to the target value and less variability. As regards acceptability, 3DP formulations prepared with different flavours and colors were well accepted by the children, although each patient had different preferences.

Personalized chewable medicines for children were produced via semisolid extrusion in the form of gummies with different shapes too, e.g., heart, bear, or disc, loaded with pediatric doses of ranitidine hydrochloride [188]. In this case, a syringe-based extrusion mechanism used mixtures of gelatin, carrageenan, xanthan gum, and sweeteners [189]. Mass as well as dose uniformity were guaranteed, fitting standards that only well-established tableting technologies can reproduce. The added value of the personalized pediatric formulations resides in the easy handling and intake, high dosage flexibility, and personalization of the final products by simple changing the size, infill density, or design of the digital model. Moreover, the attractive, funny, and appetizing appearance of the gummies could enhance treatment adherence and help to decrease the emotional impact of the disease in children.

Recently, Lego™-like chewable bricks made of edible soft material (gelatin-based matrix) were also proposed by a variant of SSE, namely embedded three-dimensional printing (e-3DP). E-3DP implies the extrusion of semisolids within a solidifying liquid matrix [190]. Such chewable dosage forms with dual drug loading (paracetamol and ibuprofen) were produced by directly extruding novel printing patterns of model drug ink (embedded phase) into a liquid gelatin-based matrix (embedding phase) at an elevated temperature (70 °C), followed by solidification at room temperature. The main advantage of this technology is the possibility to encapsulate the drug paste within a matrix that masks its flavor, as in the case of bitter-tasting drugs.

One more application of SSE is in printing lipid-based formulations (LBFs) loaded with poorly water-soluble APIs into solid oral dosage forms for the delivery of poorly water-soluble APIs. Johannesson et al., for example, used SSE to produce solid lipid tablets based on printable emulsion gels with appropriate rheological properties by the addition of methyl cellulose as viscosity enhancer [191]. Tablets loaded with fenofibrate were successfully 3D-printed, showing good mechanical and dimensional properties as well as high mass uniformity and dose accuracy. Moreover, as expected for immediate release formulations, the produced tablets were able to disintegrate in less than 15 min. The combination of the advantages of an established formulation strategy for poorly water-soluble drugs as LBFs with such a novel and flexible production technique as 3DP via SSE opens new horizons to delivery highly potent, poorly water-soluble APIs for which dose adjustments may be required in some patient categories.

ODFs (orodispersible films) may be manufactured by SSE [22,23,24]. ODFs are polymeric thin film strips loaded with drugs that rapidly dissolve upon contact with saliva [192,193]. SSE 3D printing in the production of such systems provides a very increased flexibility in terms of drug dosage as the drug dose can be established based on the dimensions of the ODF itself, representing a great advantage to realize a personalized pharmacotherapy. Yan et al. printed individualized ODFs in doses of 1.25 mg, 2.5 mg, and 5 mg of levocetirizine hydrochloride [19]. HPMC was used as water-soluble and film-forming polymer, pregelatinized starch as filling agent, and maltitol and sucralose as flavoring agents. For this kind of application, the critical point is the careful selection of the relative ratios among such components to obtain a printable ink, that is a gel with consistency and viscosity suitable for 3D printing process. Whilst the main advantage resides in the drug content uniformity and dose accuracy, high flexibility, and rapid drug release performances (complete drug dissolution within 2 min).

As FFF potential is limited by the possible thermal degradation of the APIs during the heating step, there are noteworthy examples of how the FFF-printer can be adapted for the SSE process to produce formulations while avoiding the use of high temperatures. Falcone et al. reports an example of a technological advancement fixing to the printhead of the Ultimaker3 printer a customized coaxial extruder for SSE 3D printing. The coaxial extruder was connected through tubes to the syringe pump system, allowing the simultaneous dispensing of ink gel (sodium alginate 6% *w*/*v*) and crosslinking gel (hydroxyethyl cellulose 3% *w*/*v*, calcium chloride 0.1 M and Tween 85 0.1% *v*/*v*). The innovation of this single-step approach made possible the manufacturing of innovative floating delivery systems loaded with propranolol hydrochloride and designed for personalized therapy [194].

SSE is a relevant tool in the manufacturing of medical devices (MD) as an alternative to FFF. Some interesting examples of devices produced by SSE can be found in the literature. Holländer et al., for example, applied SSE in combination with the UV-assisted crosslinking technology to produce polydimethylsiloxane (PDMS) devices for the delivery of prednisolone [26]. In this case, critical variables are the set-up of printing as well as of curing conditions, allowing the manufacture of 3D printed structures with good morphology, adequate mechanical strength, and controlled drug release.

The great potential of SEE 3DP for biomedical applications, and specifically for the manufacture of polymeric scaffolds incorporating thermolabile drugs, and hence requiring low process temperatures, has been recently evidenced. Naseri et al. reported a novel low-temperature (20 °C) 3D printing technique based on SSE poly-lactic-co-glycolic acid scaffolds using methyl ethyl ketone (MEK) as a mild organic solvent [195]. In this paper, the printability study of PLGA scaffolds was performed on different starting bio-inks obtained by varying the PLGA concentration in MEK solvent, lactic to glycolic ratio, and molecular weight of PLGA. For 3DP via SSE of PLGA scaffolds with high shape fidelity, good flexibility, and elasticity, the authors recommend PLGA concentrations higher than 80% *w*/*v*, lactic to glycolic ratio greater than 75%, molecular weight more than 100–200 kDa, and printing through nozzles smaller than 0.96 mm in internal diameter.

The same research group, starting from the above interesting results, explored semisolid extrusion 3DP in combination with the proposed and well-characterized bio-ink of PLGA to produce bioabsorbable scaffolds and, specifically, ‘biopierces’ loaded with the antibiotic mupirocin [27]. The main goal was to realize novel, bio-absorbable, drug eluting scaffolds able to cover piercing studs and prevent possible infections during the healing process. Antimicrobial sensitivity testing showed an effective release of the drug against S. aureus with an inhibition zone constant for 14 days.

More recently, drug-eluting polycaprolactone/nano-hydroxylapatite (PCL/nHA) nanocomposites loaded with vancomycin and ceftazidime and with extended drug release was fabricated using a lab-made SSE-based printer [196]. During this study, the influence of distinct printing parameters on the printed part quality was examined. The obtained results show that the tensile strength of post-printed PCL/nHA specimens increases with the fill density yet reduces with a decrease in the ratio of PCL/nHA to dichloromethane (DCM) and print speed. Dually drug loaded PCL/nHA screws were also prepared and characterized with different analytical techniques. Specifically, drug release results show for 3D printed screws an extended elution of high levels of vancomycin/ceftazidime over a 14-day period.

**Table 3:** Literature examples on dosage forms and medical devices produced via SSE 3DP. In evidence for each product—performances; —starting materials (polymers/excipients and APIs); —challenges and drawbacks.

3D Printed Product	Properties and Performances	API/s	Excipients	Challenges and Drawbacks	References
Polypill	○ Combination of five different drugs (five-in-one tablet) with two release mechanisms (sustained and immediate)	○ Pravastatin (PRV), atenolol (ATE), ramipril (RMP), acetylsalicylic acid (ASA), hydrochlorothiazide (HCT)	○ Cellulose acetate (CA), D-mannitol (MANN), PEG 6000, sodium starch glycolate (SSG) and PVP	○ The proper polypill design, material selection as well as the adequate printing sequence for producing a segmented structure with specific drug release profiles	[126]
	○ Osmotic pump and sustained release compartments	○ Captopril (CPT), nifedipine (NIF) and glipizide (GLZ)	○ Cellulose acetate (CA), D-mannitol (MANN), PEG 6000, MCC, sodium starch glycolate (SSG) and HPMC	○ To choose the proper solvent mixture (hydro-alcoholic solution) for the selected starting materials to form a paste smooth and, sufficiently consistent for printing and to better control polypill shrinkage after drying	[125]
	○ Combination of three different drug with programmed release profiles	○ Metformin hydrochloride (MET HCl), glyburide (GLB) and acarbose (ACB)	○ Pluronic F-127	○ Need for proper selection of 3D printing speed to regulate mass transfer processes during both processing and post processing intervals, guaranteeing good quality standards	[197]
	○ Tritherapeutic tablet matrix for advanced anti-HIV-1 drug delivery	○ Efavirenz (EFV), tenofovir disoproxil fumarate (TDF) and emtricitabine (EMT)	○ Brown humic acid sodium salt (HA-PQ10), hydroxyethyl cellulose ethoxylate, quaternized (QHECE) and cellulose acetate phthalate (CAP)	○ Need to enhance of HA-PQ10 sludge printability as well as its gastro-resistance. CAP was added as binder and modified-release excipient	[198]
	○ Four-in-one oral polypill with multiple release profiles	○ caffeine (CAFF) and vitamin B analogues	○ Craft Blend R30M (mixture of pharmaceutical excipients, including disintegrants and binders) and Craft Blend R4H (mixture of pharmaceutical excipients, including binders and gel forming excipients)	○ Need for selection of a proper amount of solvent to form a printable paste and avoid, at the same time, premature cogging of the nozzle tip. The optimal proportion of solvent for the paste forming allows to polypill to maintain/retain, after drying, its shape as well as mechanical integrity making it suitable for packaging, transportation, or general handling.	[199]
Immediate release tablets	○ Immediate release tablets with good content uniformity ○ The tablets with additional PVP-PVAc showed increase in dissolution and disintegration time	○ Levetiracetam (LVT)	○ PVA-PEG and PVP-PVAc)	○ To avoid loss of structural integrity after drying while maintain immediate drug release	[15]
	○ Immediate release tablets with different numbers of layer to realize different doses for pediatric subgroups	○ Levetiracetam (LVT)	○ PVA-PEG, Kollicoat^®^ IR	○ To strictly control drug content as well as mass uniformity based on the number of 3D printed layers forming the immediate release tablet	[200]
	○ Orodispersible tablets	○ Carbamazepine (CBZ)	○ Hydroxypropyl-β-cyclodextrin (HPβCD), HPMC E4M, HPMC F4M, sodium carboxymethylcellulose (CMC), PVP K25 and croscarmellose sodium (CCS);	○ To find the suitable rheological properties of 3DP-ink/paste. The adequate selection of starting materials (binder, disintegrants) as well as their relative amount/ratio, the optimal amount of wetting liquid, and the proper slicing and printing parameter set-up allow to obtain porous tablets with fast release performances and good mechanical properties	[186]
	○ Subdivided printlets for a more accurate, safe, and convenient precise hospital dispensing than traditional subdivided tablets	○ Spironolactone (SPR) and hydrochlorothiazide (HCT)	○ Lactose (L), corn starch (CS), MCC, HPMC, sucrose (Sucr) and dextrin (DX)	○ To establish a close relationship between dose and preset model for SSE-3DP	[201]
	○ Immediate release tablets with high drug loadings	○ Paracetamol (PCM)	○ Croscarmellose sodium (CCS) and PVP	○ The proper tablet design, material selection as well as drug/excipient ratio	[16]
	○ Immediate release tablets developed as three geometrical shapes (cylinder, oval and torus) and containing high amounts of drug	○ Levetiracetam (LVT)	○ Croscarmellose sodium (CCS), HPC	○ To properly select tablet geometry and architecture to enable control of drug release profiles without the need to change the paste composition (binders, disintegrants and relative mass ratio).	[12]
	○ Immediate release formulations using thermosensitive gelatin pastes	○ Ibuprofen (IBU)	○ Gelatin (Gel), glycerine (Gl), MCC, mannitol (MANN), lactose (L) and HPMC	○ To study the effect of different components and printing parameters (temperature, speed, pressure) on pastes printability and 3D printed structures deformation resistance and dissolution behavior. ○ To modulate composition and process parameters to obtain IBU immediate-release formulations with different designs	[202]
Controlled release tablets	○ Tablets with high structural integrity exhibiting sustained drug release	○ Levetiracetam (LVT)	○ PVP-PVAc, HPMC and highly dispersed silicon dioxide (SiO_2_)	○ To develop inks free of organic solvents and printable also after several days of storage ○ To optimize ink composition and digital model to obtain formulations with a sustained release of the incorporated API. (The dissolution profile could be modified by varying the amount of HPMC and by changing the infill design of tablet)	[203]
	○ Gastro-Retentive tablets	○ Ginkgolide (GNK)	○ HPMC Methocel K4M, HPMC Methocel E5LV, MCC, lactose (L), PVP K30	○ To optimize 3D printing parameters to obtain floating and accurate in shape tablets, with a satisfactory gastro-retention ability and adequate drug release profile. The optimized parameters were: full filling gap, 50%; nozzle extrusion speed, 0.006 mm/s; layer height, 0.4 mm; compensation value, 0.25; quantity of layers, 15; outline printing value, 2.)	[204]
	○ Sustained-release formulations using thermosensitive gelatin pastes	○ Diclofenac (DCF)	○ Gelatin (Gel), glycerin (Gl), MCC, mannitol (MANN), lactose (L) and HPMC	○ To study the effects of different paste components and 3D printing parameters (T, speed, pressure etc.) on printability as well as on the final performances in terms of deformation resistance and dissolution behavior of 3D printed structures.	[202]
	○ Floating drug delivery systems	○ Propranolol hydrochloride (PPN HCl)	○ sodium alginate (SAlg), CaCl_2_ and HEC	○ To simultaneously realize alginate extrusion and gelation ○ To find the optimal extrudability range of the proposed gel inks (Ink gel (SAlg 6 wt%); crosslinking gel (HEC 3 wt%, CaCl_2_ 0.1 M and Tween 85 0.1% *v*/*v*) ○ To study the impact of different digital models on drug content	[194]
Pediatric-friendly printlets	○ Chewable printlets with various flavours, colors, doses, and sizes, prepared in a hospital setting for the treatment of MSUD	○ Isoleucine (ILE)	○ Sucrose (Sucr), pectin (P) and maltodextrin (MDX)	○ To opportunely select the excipients for printing via SSE the chewable tablets (e.g., polymeric carriers, flavorings, colorants, etc.) allowing both processing as well as the good formulation acceptability by the pediatric patients. ○ To correlate tablet sizes with drug contents	[187]
	○ Gummies	○ Ranitidine hydrochloride (RN HCl)	○ Corn starch (CS), carrageenan (Carr), xanthan gum (XG), gelatin (Gel)	○ To obtain gummies with eye-catching appearance, appropriate organoleptic characteristic, and acceptable structural features, allowing easy handling and intake. ○ To optimize RN HCl release profile (release could be slowed down by varying CS amount).	[189]
	○ Gummies	○ Lamotrigine (LAM)	○ HPMC and gelatin (Gel)	○ To adjust 3DP-ink viscosity by optimizing the amount of gelatin and HPMC ○ To optimize the LAM loading within the ink (The higher the LAM content, the higher the ink viscosity)	[188]
	○ Lego™-like chewable bricks	○ Paracetamol (PCM) and ibuprofen (IBU)	○ Locust bean gum and glycerol (Gl)	○ To determine the optimal amount of APIs to load within feed solutions to obtain printable inks (formulations containing more than 40% of PCM and 28% of IBU were too viscous for efficient printing)	[190]
	○ Chocolate-based printlets in different shapes depicting cartoon characters	○ Paracetamol (PCM) and ibuprofen (IBU)	○ Bitter chocolate and corn syrup	○ To optimize corn syrup to bitter chocolate ratio (compositions with low proportions of syrup resulted in non-extrudable ink formulations; while composition with a higher syrup to chocolate ratio resulting in too sticky ink formulations that were difficult to handle)	[38]
Orodispersible films (ODFs)	○ ODFs fabricated in a one-step-process using disposable syringes	○ Warfarin (WARF)	○ HPC and PVA	○ To establish a close relationship between film sizes and drug contents ○ To find the best ink composition for 3DP process. The best print quality was gained with the 16% HPC solution at 10.4 PSI. Higher viscous solutions were printed with difficulty.	[24]
	○ Individualized ODFs	○ Levocetirizine hydrochloride (LCT HCl)	○ HPMC, pregelatinized starch (PS), maltitol (M) and sucralose (Suc)	○ To properly select ink composition to obtain ODFs with short disintegration time, good mechanical properties, and good taste (The optimal formulation was HPMC: API: PS: M: Suc at a ratio of 64:10:10:15:1) ○ To study the impact of dynamic viscosities and fluid mechanics difference on 3D printing applicability ○ To correlate theoretical model volume to drug dose	[19]
	○ Multi-layered ODFs produced with in-process drying	○ Benzydamine hydrochloride (BZY HCl)	○ Maltodextrin (MDX) (film-forming polymer) with a DE value of 5.5 (Glucidex 6–G6), Sorbitol (Sor), HEC of different viscosity grades	○ To properly select Ink composition having suitable viscosity for SSE G6 (film forming) 8 wt), Sor (plasticizer) 5 wt% in water; HEC (thickener) 1 wt% ○ To establish a good and reliable correlation between the height of digital model and weight, thickness, disintegration time and mechanical properties of prepared ODFs ○ To give the possibility to easy control the drug dose by changing the thickness respectively overall volume of digital model or the concentration of drug in the print dispersion.	[22]
	○ ODFs prepared in a hospital setting, in comparison with conventional oral formulations	○ Warfarin (WARF)	○ Lactose monohydrate (L), HPC and propylene glycol (PG)	○ To correlate weight and size of ODFs with drug content for guaranteeing uniformity and dose accuracy ○ To properly set and monitor 3D printing operative conditions (e.g., tip length, amount of ink in the syringe, pressure etc.) to produce ODFs with reproducible properties	[58]
	○ ODFs for veterinary use	○ Prednisolone (PRED)	○ PEO, HPC, pure liver powder (LP)	○ To obtain a homogenous and easily extrudable ink. Best compositions to obtain products with adequate content uniformity, immediate PRED release, high mechanical strength to withstand handling, neutral pH, and low moisture content: PRED 1 wt%; the film-forming agent HPC EXF 24–25 wt% ○ To correlate film dimensions to drug dose	[205]
Solid self-emulsifying formulations	○ Solid lipid tablets based on emulsion gels	○ Fenofibrate (FNB)	○ Maisine CC, Captex 355 EP/NF, Capmul MCM EP, Soybean oil, Kolliphor EL, Tween 85 and methyl cellulose (MC)	○ Preparation of emulsion gels with rheological properties suitable for successful 3DP leading to tablets with high mass uniformity and dose accuracy, well-defined in size, and with mechanical properties appropriate for handling. Methyl cellulose was added as viscosity	[191]
	○ Solid self-microemulsifying printlets in various geometrical shapes (i.e., cylindrical, prism, cube and torus)	○ Fenofibrate (FNB) or Cinnarizine (CNZ)	○ Gelucire^®^ 44/14, Gelucire^®^ 48/16 and Kolliphor^®^ P 188	○ Low resolution and precision respectively due to nozzle diameter and poor control of spatial distribution of the printed layers at the liquid state.	[206]
	○ Self-emulsifying suppositories	○ Tacrolimus (TC)	○ Gelucire 44/14, Gelucire 48/16 and coconut oil	○ To select the appropriate mixture of excipients to obtain a printable ink. (Gelucire44/14 or Gelucire 48/16 alone did not have adequate properties; coconut oil was employed as plasticizer to improve their performances	[207]
Medical devices	○ Hydrogel patches for local delivery of pegylated liposomal doxorubicin	○ Doxorubicin (DOX)	○ Semi-synthesized fish gelatin methacryloyl (F-GelMA), carboxymethyl cellulose sodium (CMC)	○ The optimization of hybrid hydrogel composition to obtain a printable ink F-GelMA was selected as the main component (10 wt%); CMC as thickener (1–7 wt%); liposomes as DOX carriers (10, 15 or 20 wt%) ○ To properly set post printing processing (e.g., UV exposure time) to control shape as well as DOX release	[208]
	○ Hydrogel wound dressing	○ Lidocaine hydrochloride (LDC HCl)	○ Chitosan (C) and pectin (P)	○ To prepare a hydrogel-based ink viscous enough to maintain a proper extrusion speed during 3D printing without over-liquefying or blocking in the nozzle and to conserve the geometries after printing. Being the selected material a thermoreversible hydrogel, its viscosity was adjusted by modulating the process temperature. The optimized operative conditions led to 3D printed systems with good printability, dimensional integrity, and adhesive properties.	[209]
	○ Hydrogels to treat diabetic ulcers	○ Bovine serum albumin (BSA)	○ Snakegourd root polysaccharide/Astragalus polysaccharide/CMC	○ To prepare hydrogels hard enough for SSE and properly select 3DP operative conditions for obtaining patches with uniform shape, controlled API release profiles, stiffness, and mechanical strength suitable for drug delivery applications.	[210]
	○ Bio-active patches to treat ulcers and wounds	○ Propolis (ethanolic extract)	○ High methoxylated pectin; β-Cyclodextrin (β-CD) and chitosan (C) to produce propolis inclusion complexes	○ To find the suitable ink composition allowing SSE process and the production of patches with good homogeneity, morphology, mechanical strength, and bio-adhesiveness	[211]
	○ Microneedle patches for minimally invasive glucose control in diabetes	○ Insulin (INS)	○ Sodium alginate (SAlg), hydroxyapatite (HA), CaCl_2_	○ To opportunely select bioink composition to have the rheological properties adequate for printing and guarantee shape fidelity of the 3D printed structure SAlg and HA were selected for ink preparation at 15 wt% and 8 wt%, respectively.	[212]
	○ Biopierces	○ Mupirocin (MUP)	○ PLGA	○ To eliminate organic solvent (MEK) without affecting scaffold integrity. The drying time can be further decreased using a lower vacuum pressure. However, a lower vacuum pressure increases the risk of bubble formation in the scaffold. ○ To load an optimal amount of API, ensure its integrity after processing (being sensitive to hydrolysis), and guarantee its slow release from 3D printed scaffold.	[27]
	○ Devices cured with UV light	○ Prednisolone (PRED)	○ polydimethylsiloxane (Silopren UV LSR 2030)	○ To properly select printing speed and pressure as well as post printing conditions of curing (e.g., time, intensity of the UV lamp) for hardening 3D printed structure and avoiding its collapsing ○ Need for loading an optimal amount of API to guarantee ink printability. Ink containing more than 1.5% PRED are too viscous for efficient printing	[26]
	○ PLGA/nHA scaffolds containing BMP-2 cell growth factor chitosan sustained release system to construct mandibular tissue-engineered bone	○ Recombinant human bone morphogenetic protein 2 (rhBMP-2)	○ PLGA, nHA, chitosan (C), sodium polyphosphate	○ To properly select 3DP conditions (method employed by the multi-nozzle printer, T, etc.) to realize complex scaffolds based on PLGA/nHA composite material and rhBMP-2 loaded chitosan nano sustained release carriers	[213]
	○ 3D plotted alginate fibers coated with chitosan for bone regeneration during inflammation	○ Diclofenac (DCF) and osteoblast cells	○ Sodium alginate (SAlg), CaCl_2_, chitosan (C)	○ To load an optimal amount of DCF and guarantee its slow release from 3D printed scaffold by specific post-printing treatments Coacervation with chitosan of the extruded alginate-based ink, and ionic crosslinking	[214]
+	○ Scaffolds for simultaneous local bone regeneration and infection treatments	○ Genipin (GP) and levofloxacin (LVX)	○ Biphasic calcium phosphate BCP (HA + β-TCP), chitosan (C)	○ To establish the maximum BCP loading and the optimal amount of cross-linking agent (GP) to obtain extrudable inks, with suitable rheological properties ○ The addition of levofloxacin affects ink homogeneity and scaffold porosity and mechanical strength	[215]
	○ Scaffolds	○ Dexamethasone sodium phosphate (DEXSP)	○ Gelatin (Gel) and lactose (L)	○ To properly select gelatin concentration and 3D printing parameters (T, pressure, and speed) for avoiding nozzle tip clogging as well as the spreading of the 3D printed structure on the print bed and the alteration of its shape after drying Optimized composition and operative conditions: gelatin concentration (10% *w*/*v*); T (27 °C), P (50–53 PSI), v (4 mm/s)	[216]

Abbreviations. APIs: Pravastatin (PRV); Atenolol (ATE); Ramipril (RMP); Acetylsalicylic acid (ASA); Hydrochlorothiazide (HCT); Captopril (CPT); Nifedipine (NIF); Glipizide (GLZ); Metformin hydrochloride (MET HCl); Glyburide (GLB); Acarbose (ACB); Efavirenz (EFV), Tenofovir disoproxil fumarate (TDF), Emtricitabine (EMT); Caffeine (CAFF); Levetiracetam (LVT); Carbamazepine (CBZ); Spironolactone (SPR); Hydrochlorothiazide (HCT); Paracetamol (PCM); Ibuprofen (IBU); Ginkgolide (GNK); Diclofenac (DCF); Propranolol hydrochloride (PPN HCl); Isoleucine (ILE); Ranitidine hydrochloride (RN HCl); Lamotrigine (LAM); Warfarin (WARF); Levocetirizine hydrochloride (LCT HCl); Benzydamine hydrochloride (BZY HCl); Fenofibrate (FNB); Cinnarizine (CNZ); Tacrolimus (TC); Doxorubicin (DOX); Lidocaine hydrochloride (LDC HCl); Bovine serum albumin (BSA); Insulin (INS); Mupirocin (MUP); Prednisolone (PRED); Recombinant human bone morphogenetic protein 2 (rhBMP-2); Genipin (GP); Levofloxacin (LVX); Dexamethasone sodium phosphate (DEXSP). Excipients: Cellulose acetate (CA); D-mannitol (MANN); Polyethylene glycol (PEG); Sodium Starch Glycolate (SSG); Polyvinylpyrrolidone (PVP); Brown humic acid sodium salt (HA-PQ10); hydroxyethyl cellulose ethoxylate, quaternized (QHECE); Cellulose acetate phthalate (CAP); Polyvinyl alcohol-polyethylene glycol (PVA-PEG); Polyvinylpyrrolidone-vinyl acetate copolymer (PVP-PVAc); Hydroxypropyl-β-cyclodextrin (HPβCD); Hydroxypropylmethylcellulose (HPMC), Sodium carboxymethylcellulose (CMC); Polyvinyl alcohol (PVA); Croscarmellose sodium (CCS); Lactose (L); Corn starch (CS); Microcrystalline cellulose (MCC); Sucrose (Sucr); Dextrin (DX); Sorbitol (Sor); Hydroxypropylcellulose (HPC); Hydroxyethyl cellulose (HEC); Gelatin (Gel); Glycerin (Gl); Sodium alginate (SAlg); Pectin (P); Maltodextrin (MDX); Carrageenan (Carr); Xanthan gum (XG); Pregelatinized starch (PS); Maltitol (M); Propylene glycol (PG); Silicon dioxide (SiO_2_); Poly(ethylene oxide) (PEO); Pure liver powder (LP); Methyl cellulose (MC); Chitosan (C); Hydroxyapatite (HA); Biphasic calcium phosphate (BCP); Polylactide-co-glycoside (PLGA).

### 4.3. FFF and SSE: Comparative Analysis

The product review provided in Section 4.1 and Section 4.2 confirms the high potential of extrusion-based 3DP technologies in the manufacturing of personalized drug delivery systems and medical devices, highlighting for both FFF and SSE the general advantages and disadvantages discussed early (Table 1).

Regarding FFF, filament production, as well as drug loading method, along with other formulative (e.g., starting material composition and presence of specific additives) and printing parameters (e.g., size and shape, layer height, number of overlapped layers, infill density, infill pattern, pore size, porosity, temperature of the nozzle and build platform, etc.) have a significant impact on the final performances of the 3D printed product, e.g., in terms of drug dose, release mechanism, and kinetic or mechanical strength. In more detail, physical and chemical properties of both the drug and the polymer matrix, as well as the drug loading strategy within the polymer, specifically influence the drug release mechanism (i.e., diffusion, erosion, swelling, and osmosis) [164,217,218,219,220]. For example, erosion can be predominant when the drug is loaded by impregnation within polymeric filament, while it stops to be dominant, and swelling occurs first, when the drug is loaded via HME [220]. The 3D geometric design also plays an important role in predefining and programming drug release at a defined rate, over a specified timeframe. A lot of researchers exploited FFF flexibility to vary the infill degree, and obtain an increasing or decreasing drug release rate, or a pulsatile pattern [155,156,173,181,221,222,223,224,225,226]. Another key parameter to modulate drug release kinetic is the surface area to volume ratio. Goyanes et al. [37], for example, discussed such an aspect when investigating drug release performances from different 3D printed structures (cube, pyramid, cylinder, sphere, and torus) based on eroding matrices. The geometry freedom of FFF also presents the possibility to compartmentalize the printed product without particular difficulties, obtaining, e.g., capsular devices with tailored drug combinations, doses, and release profiles [223,224,227]. Such systems can either be realized as empty compartments to be loaded with the drug later, or as outer shell and inner core in a single process [224] and allow for combinations of drugs, doses, and release kinetics [225]. The FFF critical process parameters also impact the stiffness, hardness, and mechanical strength of the final 3D printed product, aspects important for drug delivery systems (e.g., handling) [189,191,205], but even more for medical devices [85].

Differently from FFF, SSE printing uses pastes or gels. Due to the semisolid consistence of the starting materials, the main issues encountered by researchers were the nozzle clogging (due to critical rheological parameters, e.g., viscosity, yield stress under shear and compression, and viscoelastic properties) and the need for post-printing treatments (e.g., drying, cross-linking), with the following reduction of 3D printed object resolution. Material rheological properties play a crucial role in determining optimal processing conditions during SSE printing. Their careful analysis and optimization promote the homogeneous gel/paste extrusion through the nozzle, guaranteeing (1) the high reproducibility of 3D printed object; (2) the possibility to improve its mechanical properties, and (3) to control the release performances of the loaded drug [22]. As with FFF, also SSE gives high flexibility in geometry design (e.g., compartmentalization, infill degree, etc.), offering the great possibility to control drug release mechanism and kinetic. A great variety of SSE drug delivery systems, e.g., polypill [125,126,199] or floating systems [184,194,204] with unique drug release profiles, were produced by simply varying the design and printing parameters. In addition, the possibility to work with a wide range of excipients of well-established use in the pharmaceutical field, and at room temperature (suitable for thermolabile APIs and excipients), highly increases the application domain of such a 3DP approach in this sector. In fact, SSE has been highly investigated to produce immediate release tablets [12,16,203] or orodispersible films [19,22,186], using traditional super disintegrants and other excipients as ink components.

In general, both extrusion-based 3DP techniques FFF and SSE allow the preparation of complex structures with high control over the pore size, pore architecture and wall thickness, exposed surface area, and pore interconnectivity, key factors to control drug release profiles, mechanical strength, and other critical products, guaranteeing the successful production of personalized drug delivery systems as well as medical devices. However, their pertinency is not overlapping due to the respective disadvantages that limit some specific applications. Using one technique or the other depends on the starting materials used as well as on the final performances of the 3D printed products.

## 5. Conclusions

The technology evolution pathway of 3DP from 2012 to date is very notable. It is a fact that at present there is a contribution of 3D-printing to many aspects of healthcare. However, the full range of application remains to be explored in depth.

In the last few years, the healthcare needs of the population have changed, also thanks to the adoption and enhancement of omics technologies in healthcare. There is an increasing demand of patient-tailored treatments to improve efficacy, safety, patient compliance, therapeutic adherence, as well as cost-efficiency. This has strongly moved the attention towards 3D printing technology, which offers innovative, digitally designed solutions able to overcome the issues of the currently marketed traditional products.

Problems impacting 3DP application are mainly four-fold: the strict requirements for excipients, the development of printing software and equipment, the optimization of mechanical properties of products, and the regulatory framework. As regards excipients for pharmaceutical 3D printing, they are relatively restricted, compared to conventional manufacturing processes, mainly for technologies using heat. Much research has updated this field. However, further studies concerning biocompatible, biodegradable, and stable excipients peculiar for 3DP are required. As the complexity of product structure increases, the modeling and slicing software used to design and drive its manufacture as well as equipment, operative procedures, and control system must be constantly refreshed.

From a regulatory point of view, there are still several open questions surrounding how 3D-printed healthcare products can be monitored and evaluated for quality. Although the FDA authorized in 2015 the first 3D-printed tablets, no regulations or guidelines regarding 3D-printed medicines are currently available. Progress has been made for 3D printed medical devices, for which the FDA released in 2017 guidance detailing some technical considerations. However, there is no possibility to give universal guidelines for all 3D-printed technologies and medical devices. A separate assessment of safety and effectiveness may be required for each technology and product, especially for those personalized. Furthermore, when products are customized to the patient, the question of whether 3D printing is classed as a manufacturing or compounding process also has a great impact on regulatory requirements.

The present review was addressed to show how 3D printing (3DP) can develop in the next years focusing on the identification of areas where current research is more pro-active and tentatively providing directions for future research. Particularly, given the implementation of existing technologies, the investigation of new or re-designed materials and apparatus appears necessary for arriving at decentralized and customized digital manufacturing perfectly integrated into manufacturing systems for medicines and medical devices.

Overall, 3D printing has the potential to revolutionize the existing healthcare scenario, allowing not only the development of new materials and drug delivery systems, but also the choice between centralized/decentralized manufacturing, customized/personalized medicines and medical devices, and on demand/by market order request production. However, we will have to wait and see whether the technical and regulatory challenges facing the market can be overcome, enabling the technology to reach its full therapeutic potential.

## Figures and Tables

**Table 1:** Overview on advantages and limitations of the main 3D Printing technologies analyzed.

Method	Materials		Advantages	Disadvantages
Ink-jet 3DP	CIJ	Drug solution as ink and edible sheet as substrate	▪ Limited clogging of nozzle	▪ Wastage of material ▪ Low resolution ▪ Expensive
	DOD	Wax and ceramics	▪ Instantaneous solidification ▪ Use of small droplet volume permits high-resolution printing ▪ High efficiency ▪ Cost-effectiveness ▪ Minimal wastage of material	▪ Low selection of starting materials ▪ Need for supporting structures for overhang geometries
	DOS	Binder fluid and powder bed which can be composed by most excipients used in pharmaceutical technology, e.g., starch (S), lactose (L), maltitol (M), maltodextrin (MDX), HPMC, PVP, etc.	▪ Low cost ▪ Large-scale room temperature manufacturing process ▪ Being a low temperature process, it is suitable for thermolabile drugs) ▪ Fast production ▪ Precise location of exact dose of drug or excipients within powdered bed to obtain several compartments with different composition or mode of action ▪ Large choice of starting materials (powders and binder solutions) ▪ Multi-material printing ▪ Need for supporting structures ▪ Possibility to produce more porous structure as compared to conventional tableting with fast disintegration time ▪ Recycled raw materials	▪ Use of organic solvent ▪ Critical properties of printing fluid ▪ Need for post-processing (e.g., drying to remove residual solvents and improve physical resistance; unbound powder removal to eliminate excess powders accumulated during printing) ▪ Poor mechanical resistance and high friability of the final dosage form ▪ Low material utilization ▪ Powder wastage requiring a specialized powder facility
Laser based 3DP	SLS	Laser energy absorbing powders, e.g., PVA-PEG, PC, PE, etc.	▪ Solvent-free process ▪ Easy to use ▪ High resolution, precision, and accuracy ▪ High surface finish ▪ Good mechanical properties ▪ Design freedom ▪ No need for supporting structures ▪ Highly controllable internal microstructures ▪ Recycled raw materials	▪ Expensive ▪ Only laser energy absorbing components can be used ▪ Suitable particle size for powder bed ▪ Limited speed for sintering ▪ Need for post-processing (after printing the fabricated object are embedded in powder and the bed should be slowly cooled down to avoid stress) ▪ High energy input (risk of degradation of drugs and excipients) ▪ Wastage of unsintered powder
	SLA	Photo-curable liquid resins, e.g., PEGDA, PEG, PEG-DMA, pHEMA, PPF/DEF, etc.	▪ Easy to use ▪ Ability to fabricate submicron-sized objects and micro-sized layers ▪ Large parts can be built easily with a resolution down to 0.2 micron ▪ Very high resolution, accuracy, and surface finish ▪ Wide range of applications	▪ Costly equipment ▪ Long printing time ▪ Low efficiency ▪ Few resins effectively usable ▪ Need for supporting structures ▪ Need for post-processing (to further cure the final product; to improve its mechanical integrity and to polish or remove the attached supports to the fabricated object) ▪ Potential material toxicity (few polymers approved for pharmaceutical use) ▪ Long-term stability issues ▪ Limited material selection (UV-curable substances) ▪ Not well-defined mechanical properties due to the usage of RESINmers (such properties can decrease over time)
Nozzle based 3DP	FFF	Thermoplastic polymers (mainly in form of filaments), e.g., PVA, PLA, PLGA, PCL, TCP, HPC, Eudragit, HPMCAS, Soluplus^®^, etc.	▪ Cheap, widely available, compact, and easy to use equipment ▪ High speed ▪ Medium resolution ▪ Very good accuracy ▪ Good mechanical properties ▪ High quality ▪ High drug uniformity ▪ No need for post-printing ▪ Optimum in term of design complexity ▪ Used for a wide range of thermoplastic materials	▪ Poor surface finish (rough surfaces) ▪ Need for supporting structures (depending on printed geometry) ▪ High temperature process (potential risk of thermal degradation for drug/s and excipients) ▪ Limited material selection (thermoplastic polymers) ▪ Need for filament fabrication, as well as drug loading in a previous step (except when using Direct Powder Extrusion 3D printer), with a general increase of production costs ▪ Low drug loading ▪ Difficult to scale up
	SSE	Semisolid mixture of polymers and solvents It allows the use of the most types of excipients used in pharmaceutical technology such as HPMC, HPC, PVP, MCC, etc.	▪ Cheap, readily available, and easy to use ▪ Low temperature process (suitable for thermolabile drugs) ▪ High drug loading (up to 90%) ▪ Large choice of starting materials ▪ Multi-material printing ▪ Ability to manufacture drug loaded devices with multi-release modo	▪ Use of organic solvents ▪ Limited resolution (depending on nozzle size) ▪ Need for post-processing (e.g., drying) ▪ Low efficiency ▪ Low mechanical properties (low hardness and high friability) ▪ Need for sufficiently viscous semi-solid materials ▪ Difficulty to control the flow of semisolid materials through the nozzle ▪ Risk of nozzle clogging

Abbreviations. Starch (S); Lactose (L); Maltitol (M); Maltodextrin (MDX); Hydroxypropylmethylcellulose (HPMC); Polyvinylpyrrolidone (PVP); polyvinyl alcohol-polyethylene glycol (PVA-PEG); Polycarbonate (PC); polyethylene (PE); poly(ethylene glycol) diacrylate (PEGDA); polyethylene glycol (PEG); poly(ethylene glycol) dimethacrylate (PEG-DMA); poly(2-hydroxyethyl methacrylate) (pHEMA); poly(propylene fumarate)/diethyl fumarate (PPF/DEF); polyvinyl alcohol (PVA), Poly(lactic acid) (PLA), polylactide-co-glycoside (PLGA); Polycaprolactone (PCL); Tribasic calcium phosphate (TCP); Hydroxypropylcellulose (HPC); Poly (methyl methacrylate) derivatives, Eudragit^®^ (E); Hydroxyl propyl methyl cellulose acetate succinate (HPMCAS); Polyvinyl caprolactam-polyvinyl acetate-polyethylene glycol graft copolymer, Soluplus^®^ (SLP); Microcrystalline cellulose (MCC).

## Data Availability

Not applicable.

## References

[1] Jamróz W., Szafraniec J., Kurek M., Jachowicz R. (2018). 3D Printing in Pharmaceutical and Medical Applications-Recent Achievements and Challenges. Pharm. Res..

[2] Gao W., Zhang Y., Ramanujan D., Ramani K., Chen Y., Williams C.B., Wang C.C., Shin Y.C., Zhang S., Zavattieri P.D. (2015). The status, challenges, and future of additive manufacturing in engineering. Comput.-Aided Des..

[3] Di Prima M., Coburn J., Hwang D., Kelly J., Khairuzzaman A., Ricles L. (2016). Additively manufactured medical product–the FDA perspective. 3d Print. Med..

[4] West T.G., Bradbury T.J. (2019). 3D Printing: A Case of ZipDose^®^ Technology–World’s First 3D Printing Platform to Obtain FDA Approval for a Pharmaceutical Product. 3D and 4D Printing in Biomedical Applications: Process Engineering and Additive Manufacturing.

[5] Norman J., Madurawe R.D., Moore C.M., Khan M.A., Khairuzzaman A. (2017). A new chapter in pharmaceutical manufacturing: 3D-printed drug products. Adv. Drug Deliv. Rev..

[6] Eshkalak S.K., Ghomi E.R., Dai Y., Choudhury D., Ramakrishna S. (2020). The role of three-dimensional printing in healthcare and medicine. Mater. Des..

[7] Jose P.A., GV P.C. (2018). 3D printing of pharmaceuticals–a potential technology in developing personalized medicine. Asian J. Pharm. Res. Dev..

[8] Ong J.J., Awad A., Martorana A., Gaisford S., Stoyanov E., Basit A.W., Goyanes A. (2020). 3D printed opioid medicines with alcohol-resistant and abuse-deterrent properties. Int. J. Pharm..

[9] Hamed R., Mohamed E.M., Rahman Z., Khan M.A. (2021). 3D-printing of lopinavir printlets by selective laser sintering and quantification of crystalline fraction by XRPD-chemometric models. Int. J. Pharm..

[10] Mohamed E.M., Ali S.F.B., Rahman Z., Dharani S., Ozkan T., Kuttolamadom M.A., Khan M.A. (2020). Formulation optimization of selective laser sintering 3D-printed tablets of clindamycin palmitate hydrochloride by response surface methodology. AAPS PharmSciTech.

[11] Fang D., Yang Y., Cui M., Pan H., Wang L., Li P., Wu W., Qiao S., Pan W. (2021). Three-Dimensional (3D)–Printed Zero-Order Released Platform: A Novel Method of Personalized Dosage Form Design and Manufacturing. AAPS PharmSciTech.

[12] Cui M., Pan H., Fang D., Qiao S., Wang S., Pan W. (2020). Fabrication of high drug loading levetiracetam tablets using semi-solid extrusion 3D printing. J. Drug Deliv. Sci. Technol..

[13] Goyanes A., Buanz A.B., Hatton G.B., Gaisford S., Basit A.W. (2015). 3D printing of modified-release aminosalicylate (4-ASA and 5-ASA) tablets. Eur. J. Pharm. Biopharm..

[14] Solanki N.G., Tahsin M., Shah A.V., Serajuddin A.T. (2018). Formulation of 3D printed tablet for rapid drug release by fused deposition modeling: Screening polymers for drug release, drug-polymer miscibility and printability. J. Pharm. Sci..

[15] El Aita I., Breitkreutz J., Quodbach J. (2019). On-demand manufacturing of immediate release levetiracetam tablets using pressure-assisted microsyringe printing. Eur. J. Pharm. Biopharm..

[16] Khaled S.A., Alexander M.R., Wildman R.D., Wallace M.J., Sharpe S., Yoo J., Roberts C.J. (2018). 3D extrusion printing of high drug loading immediate release paracetamol tablets. Int. J. Pharm..

[17] Matijašić G., Gretić M., Vinčić J., Poropat A., Cuculić L., Rahelić T. (2019). Design and 3D printing of multi-compartmental PVA capsules for drug delivery. J. Drug Deliv. Sci. Technol..

[18] Azizi Machekposhti S., Mohaved S., Narayan R.J. (2019). Inkjet dispensing technologies: Recent advances for novel drug discovery. Expert Opin. Drug Discov..

[19] Yan T.-T., Lv Z.-F., Tian P., Lin M.-M., Lin W., Huang S.-Y., Chen Y.-Z. (2020). Semi-solid extrusion 3D printing ODFs: An individual drug delivery system for small scale pharmacy. Drug Dev. Ind. Pharm..

[20] Musazzi U.M., Selmin F., Ortenzi M.A., Mohammed G.K., Franzé S., Minghetti P., Cilurzo F. (2018). Personalized orodispersible films by hot melt ram extrusion 3D printing. Int. J. Pharm..

[21] Jamróz W., Kurek M., Łyszczarz E., Szafraniec J., Knapik-Kowalczuk J., Syrek K., Paluch M., Jachowicz R. (2017). 3D printed orodispersible films with Aripiprazole. Int. J. Pharm.

[22] Elbl J., Gajdziok J., Kolarczyk J. (2020). 3D printing of multilayered orodispersible films with in-process drying. Int. J. Pharm..

[23] Tian Y., Orlu M., Woerdenbag H.J., Scarpa M., Kiefer O., Kottke D., Sjöholm E., Öblom H., Sandler N., Hinrichs W.L.J. (2019). Oromucosal films: From patient centricity to production by printing techniques. Expert Opin. Drug Deliv..

[24] Sjöholm E., Sandler N. (2019). Additive manufacturing of personalized orodispersible warfarin films. Int. J. Pharm..

[25] Tiboni M., Campana R., Frangipani E., Casettari L. (2021). 3D printed clotrimazole intravaginal ring for the treatment of recurrent vaginal candidiasis. Int. J. Pharm..

[26] Holländer J., Hakala R., Suominen J., Moritz N., Yliruusi J., Sandler N. (2018). 3D printed UV light cured polydimethylsiloxane devices for drug delivery. Int. J. Pharm..

[27] Naseri E., Cartmell C., Saab M., Kerr R.G., Ahmadi A. (2020). Development of 3D printed drug-eluting scaffolds for preventing piercing infection. Pharmaceutics.

[28] Choonara Y.E., du Toit L.C., Kumar P., Kondiah P.P., Pillay V. (2016). 3D-printing and the effect on medical costs: A new era?. Expert Rev. Pharm. Outcomes Res..

[29] Choi W.J., Hwang K.S., Kwon H.J., Lee C., Kim C.H., Kim T.H., Heo S.W., Kim J.-H., Lee J.-Y. (2020). Rapid development of dual porous poly(lactic acid) foam using fused deposition modeling (FDM) 3D printing for medical scaffold application. Mater. Sci. Eng. C.

[30] Awad A., Trenfield S.J., Goyanes A., Gaisford S., Basit A.W. (2018). Reshaping drug development using 3D printing. Drug Discov. Today.

[31] Mathew E., Pitzanti G., Larrañeta E., Lamprou D.A. (2020). 3D Printing of Pharmaceuticals and Drug Delivery Devices.

[32] Report G. (2022). 3D Printed Drugs Market Research Report by Technology, by Region–Global Forecast to 2025–Cumulative Impact of COVID-19.

[33] Everett H. (2021). Triastek Receives FDA IND Clearance for 3D Printed Drug to Treat Rheumatoid Arthritis.

[34] Adnkronos Triastek Closes US$ 50 Million Series B Financing, Co-led by Matrix Partners China and CPE. https://www.adnkronos.com/triastek-closes-us-50-million-series-b-financing-co-led-by-matrix-partners-china-and-cpe_3q9ZwFgfwSifRAQVbQn6Qi.

[35] Sadia M., Arafat B., Ahmed W., Forbes R.T., Alhnan M.A. (2018). Channelled tablets: An innovative approach to accelerating drug release from 3D printed tablets. J. Control. Release.

[36] Li Q., Wen H., Jia D., Guan X., Pan H., Yang Y., Yu S., Zhu Z., Xiang R., Pan W. (2017). Preparation and investigation of controlled-release glipizide novel oral device with three-dimensional printing. Int. J. Pharm..

[37] Goyanes A., Robles Martinez P., Buanz A., Basit A.W., Gaisford S. (2015). Effect of geometry on drug release from 3D printed tablets. Int. J. Pharm..

[38] Karavasili C., Gkaragkounis A., Moschakis T., Ritzoulis C., Fatouros D.G. (2020). Pediatric-friendly chocolate-based dosage forms for the oral administration of both hydrophilic and lipophilic drugs fabricated with extrusion-based 3D printing. Eur. J. Pharm. Sci..

[39] Kyobula M., Adedeji A., Alexander M.R., Saleh E., Wildman R., Ashcroft I., Gellert P.R., Roberts C.J. (2017). 3D inkjet printing of tablets exploiting bespoke complex geometries for controlled and tuneable drug release. J. Control. Release.

[40] Wang J., Goyanes A., Gaisford S., Basit A.W. (2016). Stereolithographic (SLA) 3D printing of oral modified-release dosage forms. Int. J. Pharm..

[41] Pere C.P.P., Economidou S.N., Lall G., Ziraud C., Boateng J.S., Alexander B.D., Lamprou D.A., Douroumis D. (2018). 3D printed microneedles for insulin skin delivery. Int. J. Pharm..

[42] Goyanes A., Det-Amornrat U., Wang J., Basit A.W., Gaisford S. (2016). 3D scanning and 3D printing as innovative technologies for fabricating personalized topical drug delivery systems. J. Control. Release.

[43] Vivero-Lopez M., Xu X., Muras A., Otero A., Concheiro A., Gaisford S., Basit A.W., Alvarez-Lorenzo C., Goyanes A. (2021). Anti-biofilm multi drug-loaded 3D printed hearing aids. Mater. Sci. Eng. C.

[44] Andrés-Cano P., Calvo-Haro J.A., Fillat-Gomà F., Andrés-Cano I., Perez-Mañanes R. (2021). Role of the orthopaedic surgeon in 3D printing: Current applications and legal issues for a personalized medicine. Rev. Española De Cirugía Ortopédica Y Traumatol. (Engl. Ed. ).

[45] Fu J., Yu X., Jin Y. (2018). 3D printing of vaginal rings with personalized shapes for controlled release of progesterone. Int. J. Pharm..

[46] Jung J.P., Bhuiyan D.B., Ogle B.M. (2016). Solid organ fabrication: Comparison of decellularization to 3D bioprinting. Biomater. Res..

[47] Sonova. https://www.sonova.com/en.

[48] Beer N., Hegger I., Kaae S., De Bruin M.L., Genina N., Alves T.L., Hoebert J., Kälvemark Sporrong S. (2021). Scenarios for 3D printing of personalized medicines-A case study. Explor. Res. Clin. Soc. Pharm..

[49] Beg S., Almalki W.H., Malik A., Farhan M., Aatif M., Rahman Z., Alruwaili N.K., Alrobaian M., Tarique M., Rahman M. (2020). 3D printing for drug delivery and biomedical applications. Drug Discov. Today.

[50] Flores M., Glusman G., Brogaard K., Price N.D., Hood L. (2013). P4 medicine: How systems medicine will transform the healthcare sector and society. Per Med..

[51] Mathur S., Sutton J. (2017). Personalized medicine could transform healthcare. Biomed. Rep..

[52] Goole J., Amighi K. (2016). 3D printing in pharmaceutics: A new tool for designing customized drug delivery systems. Int. J. Pharm.

[53] Afsana, Jain V., Haider N., Jain K. (2018). 3D Printing in Personalized Drug Delivery. Curr Pharm Des..

[54] Vaz V.M., Kumar L. (2021). 3D printing as a promising tool in personalized medicine. AAPS PharmSciTech.

[55] Arefin A.M., Khatri N.R., Kulkarni N., Egan P.F. (2021). Polymer 3D printing review: Materials, process, and design strategies for medical applications. Polymers.

[56] Robles-Martinez P., Xu X., Trenfield S.J., Awad A., Goyanes A., Telford R., Basit A.W., Gaisford S. (2019). 3D printing of a multi-layered polypill containing six drugs using a novel stereolithographic method. Pharmaceutics.

[57] Lafeber I., Ruijgrok E.J., Guchelaar H.-J., Schimmel K.J.M. (2022). 3D Printing of Pediatric Medication: The End of Bad Tasting Oral Liquids?—A Scoping Review. Pharmaceutics.

[58] Öblom H., Sjöholm E., Rautamo M., Sandler N. (2019). Towards Printed Pediatric Medicines in Hospital Pharmacies: Comparison of 2D and 3D-Printed Orodispersible Warfarin Films with Conventional Oral Powders in Unit Dose Sachets. Pharmaceutics.

[59] Martinez P.R., Goyanes A., Basit A.W., Gaisford S. (2017). Fabrication of drug-loaded hydrogels with stereolithographic 3D printing. Int. J. Pharm..

[60] Varghese R., Salvi S., Sood P., Karsiya J., Kumar D. (2022). 3D printed medicine for the management of chronic diseases: The road less travelled. Ann. 3d Print. Med..

[61] Leite M., Soares B., Lopes V., Santos S., Silva M.T. (2019). Design for personalized medicine in orthotics and prosthetics. Procedia CIRP.

[62] van der Stelt M., Grobusch M.P., Koroma A.R., Papenburg M., Kebbie I., Slump C.H., Maal T.J.J., Brouwers L. (2021). Pioneering low-cost 3D-printed transtibial prosthetics to serve a rural population in Sierra Leone—An observational cohort study. EClinicalMedicine.

[63] Fan H., Fu J., Li X., Pei Y., Li X., Pei G., Guo Z. (2015). Implantation of customized 3-D printed titanium prosthesis in limb salvage surgery: A case series and review of the literature. World J. Surg. Oncol..

[64] Zhu Y., Liu K., Deng J., Ye J., Ai F., Ouyang H., Wu T., Jia J., Cheng X., Wang X. (2019). 3D printed zirconia ceramic hip joint. with precise structure and broad-spectrum antibacterial properties. Int. J. Nanomed..

[65] Sheela U.B., Usha P.G., Joseph M.M., Melo J.S., Thankappan Nair S.T., Tripathi A., Thomas D.J., Singh D. (2021). 7-3D printing in dental implants. 3D Printing in Medicine and Surgery.

[66] Prakash D., Davis R., Sharma A.K. (2019). Design and Fabrication of Dental Implant Prototypes Using Additive Manufacturing. First International Conference on Materials Science and Manufacturing Technology.

[67] Calvo-Haro J.A., Pascau J., Mediavilla-Santos L., Sanz-Ruiz P., Sánchez-Pérez C., Vaquero-Martín J., Perez-Mañanes R. (2021). Conceptual evolution of 3D printing in orthopedic surgery and traumatology: From “do it yourself” to “point of care manufacturing”. BMC Musculoskelet. Disord..

[68] Wixted C.M., Peterson J.R., Kadakia R.J., Adams S.B. (2021). Three-dimensional Printing in Orthopaedic Surgery: Current Applications and Future Developments. JAAOS Glob. Res. Rev..

[69] Wu Y., Kennedy P., Bonazza N., Yu Y., Dhawan A., Ozbolat I. (2021). Three-Dimensional Bioprinting of Articular Cartilage: A Systematic Review. Cartilage.

[70] Xiongfa J., Hao Z., Liming Z., Jun X. (2018). Recent advances in 3D bioprinting for the regeneration of functional cartilage. Regen. Med..

[71] Agarwal S., Saha S., Balla V.K., Pal A., Barui A., Bodhak S. (2020). Current Developments in 3D Bioprinting for Tissue and Organ Regeneration–a Review. Front. Mech. Eng..

[72] Weisman J.A., Ballard D.H., Jammalamadaka U., Tappa K., Sumerel J., D’Agostino H.B., Mills D.K., Woodard P.K. (2019). 3D Printed Antibiotic and Chemotherapeutic Eluting Catheters for Potential Use in Interventional Radiology: In Vitro Proof of Concept Study. Acad. Radiol..

[73] Kim T.H., Lee J.-H., Ahn C.B., Hong J.H., Son K.H., Lee J.W. (2019). Development of a 3D-Printed Drug-Eluting Stent for Treating Obstructive Salivary Gland Disease. ACS Biomater. Sci. Eng..

[74] Tappa K., Jammalamadaka U., Weisman J.A., Ballard D.H., Wolford D.D., Pascual-Garrido C., Wolford L.M., Woodard P.K., Mills D.K. (2019). 3D printing custom bioactive and absorbable surgical screws, pins, and bone plates for localized drug delivery. J. Funct. Biomater..

[75] Domsta V., Seidlitz A. (2021). 3D-Printing of Drug-Eluting Implants: An Overview of the Current Developments Described in the Literature. Molecules.

[76] Wang Z., Yang Y. (2021). Application of 3D Printing in Implantable Medical Devices. BioMed Res. Int..

[77] Mohamdeen Y.M.G., Tabriz A.G., Tighsazzadeh M., Nandi U., Khalaj R., Andreadis I., Boateng J.S., Douroumis D. (2021). Development of 3D printed drug-eluting contact lenses. J. Pharm Pharm..

[78] Beitler B.G., Abraham P.F., Glennon A.R., Tommasini S.M., Lattanza L.L., Morris J.M., Wiznia D.H. (2022). Interpretation of regulatory factors for 3D printing at hospitals and medical centers, or at the point of care. 3d Print. Med..

[79] ASME 3D Printing Medical Devices at the Point of Care: Webinar Series. https://resources.asme.org/poc3dp-events.

[80] GOV.UK Consultation on Point of Care Manufacturing. https://www.gov.uk/government/consultations/point-of-care-consultation/consultation-on-point-of-care-manufacturing.

[81] Boon W., van Wee B. (2018). Influence of 3D printing on transport: A theory and experts judgment based conceptual model. Transp. Rev..

[82] Manners-Bell J., Lyon K. (2012). The implications of 3D printing for the global logistics industry. Transp. Intell..

[83] Campbell T., Williams C., Ivanova O., Garrett B. (2011). Could 3D printing change the world. Technologies, Potential, and Implications of Additive Manufacturing.

[84] Zhou H., Bhaduri S.B., Yang L., Bhaduri S.B., Webster T.J. (2019). 12-3D printing in the research and development of medical devices. Biomaterials in Translational Medicine.

[85] Durfee W.K., Iaizzo P.A., Iaizzo P.A. (2019). Chapter 21-Medical Applications of 3D Printing. Engineering in Medicine.

[86] Morrison R.J., Kashlan K.N., Flanangan C.L., Wright J.K., Green G.E., Hollister S.J., Weatherwax K.J. (2015). Regulatory Considerations in the Design and Manufacturing of ImplanTable 3D-Printed Medical Devices. Clin. Transl. Sci..

[87] FDA (2017). Technical Considerations for Additive Manufactured Medical Devices. FDA Center for Devices and Radiological Health.

[88] Ian Gibson I.G. (2015). Additive Manufacturing Technologies 3D Printing, Rapid Prototyping, and Direct Digital Manufacturing.

[89] (2021). Additive Manufacturing—General Principles—Fundamentals and Vocabulary.

[90] Saleh Alghamdi S., John S., Roy Choudhury N., Dutta N.K. (2021). Additive Manufacturing of Polymer Materials: Progress, Promise and Challenges. Polymers.

[91] Mohapatra S., Kar R.K., Biswal P.K., Bindhani S. (2022). Approaches of 3D printing in current drug delivery. Sens. Int..

[92] Barui S. (2021). 3D inkjet printing of biomaterials: Principles and applications. Med. Devices Sens..

[93] Hsiao W.-K., Lorber B., Reitsamer H., Khinast J. (2018). 3D printing of oral drugs: A new reality or hype?. Expert Opin. Drug Deliv..

[94] Karalia D., Siamidi A., Karalis V., Vlachou M. (2021). 3D-Printed oral dosage forms: Mechanical properties, computational approaches and applications. Pharmaceutics.

[95] van den Heuvel K.A., de Wit M.T.W., Dickhoff B.H.J. (2021). Evaluation of lactose based 3D powder bed printed pharmaceutical drug product tablets. Powder Technol..

[96] Wilts E.M., Ma D., Bai Y., Williams C.B., Long T.E. (2019). Comparison of linear and 4-arm star poly (vinyl pyrrolidone) for aqueous binder jetting additive manufacturing of personalized dosage tablets. ACS Appl. Mater. Interfaces.

[97] Infanger S., Haemmerli A., Iliev S., Baier A., Stoyanov E., Quodbach J. (2019). Powder bed 3D-printing of highly loaded drug delivery devices with hydroxypropyl cellulose as solid binder. Int. J. Pharm..

[98] Tian P., Yang F., Yu L.-P., Lin M.-M., Lin W., Lin Q.-F., Lv Z.-F., Huang S.-Y., Chen Y.-Z. (2019). Applications of excipients in the field of 3D printed pharmaceuticals. Drug Dev. Ind. Pharm..

[99] Yu D.-G., Branford-White C., Ma Z.-H., Zhu L.-M., Li X.-Y., Yang X.-L. (2009). Novel drug delivery devices for providing linear release profiles fabricated by 3DP. Int. J. Pharm..

[100] Shi K., Tan D.K., Nokhodchi A., Maniruzzaman M. (2019). Drop-on-powder 3D printing of tablets with an anti-cancer drug, 5-fluorouracil. Pharmaceutics.

[101] Sen K., Manchanda A., Mehta T., Ma A.W., Chaudhuri B. (2020). Formulation design for inkjet-based 3D printed tablets. Int. J. Pharm..

[102] Charoo N.A., Barakh Ali S.F., Mohamed E.M., Kuttolamadom M.A., Ozkan T., Khan M.A., Rahman Z. (2020). Selective laser sintering 3D printing–an overview of the technology and pharmaceutical applications. Drug Dev. Ind. Pharm..

[103] Awad A., Fina F., Goyanes A., Gaisford S., Basit A.W. (2020). 3D printing: Principles and pharmaceutical applications of selective laser sintering. Int. J. Pharm..

[104] Kafle A., Luis E., Silwal R., Pan H.M., Shrestha P.L., Bastola A.K. (2021). 3D/4D Printing of polymers: Fused deposition modelling (FDM), selective laser sintering (SLS), and stereolithography (SLA). Polymers.

[105] Fina F., Madla C.M., Goyanes A., Zhang J., Gaisford S., Basit A.W. (2018). Fabricating 3D printed orally disintegrating printlets using selective laser sintering. Int. J. Pharm..

[106] Fina F., Goyanes A., Gaisford S., Basit A.W. (2017). Selective laser sintering (SLS) 3D printing of medicines. Int. J. Pharm..

[107] Low K., Leong K., Chua C., Du Z., Cheah C. (2001). Characterization of SLS parts for drug delivery devices. Rapid Prototyp. J..

[108] Cheah C., Leong K., Chua C., Low K., Quek H. (2002). Characterization of microfeatures in selective laser sintered drug delivery devices. Proc. Inst. Mech. Eng. Part H J. Eng. Med..

[109] Trenfield S.J., Goyanes A., Telford R., Wilsdon D., Rowland M., Gaisford S., Basit A.W. (2018). 3D printed drug products: Non-destructive dose verification using a rapid point-and-shoot approach. Int. J. Pharm..

[110] Fina F., Goyanes A., Madla C.M., Awad A., Trenfield S.J., Kuek J.M., Patel P., Gaisford S., Basit A.W. (2018). 3D printing of drug-loaded gyroid lattices using selective laser sintering. Int. J. Pharm..

[111] Allahham N., Fina F., Marcuta C., Kraschew L., Mohr W., Gaisford S., Basit A.W., Goyanes A. (2020). Selective laser sintering 3D printing of orally disintegrating printlets containing ondansetron. Pharmaceutics.

[112] Vithani K., Goyanes A., Jannin V., Basit A.W., Gaisford S., Boyd B.J. (2019). An overview of 3D printing technologies for soft materials and potential opportunities for lipid-based drug delivery systems. Pharm. Res..

[113] Chia H.N., Wu B.M. (2015). Recent advances in 3D printing of biomaterials. J. Biol. Eng..

[114] Quan H., Zhang T., Xu H., Luo S., Nie J., Zhu X. (2020). Photo-curing 3D printing technique and its challenges. Bioact. Mater..

[115] Martinez P.R., Goyanes A., Basit A.W., Gaisford S. (2018). Influence of geometry on the drug release profiles of stereolithographic (SLA) 3D-printed tablets. AAPS PharmSciTech.

[116] Karakurt I., Aydoğdu A., Çıkrıkcı S., Orozco J., Lin L. (2020). Stereolithography (SLA) 3D printing of ascorbic acid loaded hydrogels: A controlled release study. Int. J. Pharm..

[117] Konasch J., Riess A., Mau R., Teske M., Rekowska N., Eickner T., Grabow N., Seitz H. (2019). A novel hybrid additive manufacturing process for drug delivery systems with locally incorporated drug depots. Pharmaceutics.

[118] Forouzandeh F., Ahamed N.N., Hsu M.-C., Walton J.P., Frisina R.D., Borkholder D.A. (2020). A 3D-printed modular microreservoir for drug delivery. Micromachines.

[119] Park B.J., Choi H.J., Moon S.J., Kim S.J., Bajracharya R., Min J.Y., Han H.-K. (2019). Pharmaceutical applications of 3D printing technology: Current understanding and future perspectives. J. Pharm. Investig..

[120] Awad A., Trenfield S.J., Gaisford S., Basit A.W. (2018). 3D printed medicines: A new branch of digital healthcare. Int. J. Pharm..

[121] Alhijjaj M., Belton P., Qi S. (2016). An investigation into the use of polymer blends to improve the printability of and regulate drug release from pharmaceutical solid dispersions prepared via fused deposition modeling (FDM) 3D printing. Eur. J. Pharm. Biopharm..

[122] Tan L.J., Zhu W., Zhou K. (2020). Recent Progress on Polymer Materials for Additive Manufacturing. Adv. Funct. Mater..

[123] Elbadawi M., McCoubrey L.E., Gavins F.K., Ong J.J., Goyanes A., Gaisford S., Basit A.W. (2021). Harnessing Artificial Intelligence for the Next Generation of 3D Printed Medicines. Adv. Drug Deliv. Rev..

[124] Basit A.W., Gaisford S. (2018). 3D Printing of Pharmaceuticals.

[125] Khaled S.A., Burley J.C., Alexander M.R., Yang J., Roberts C.J. (2015). 3D printing of tablets containing multiple drugs with defined release profiles. Int. J. Pharm.

[126] Khaled S.A., Burley J.C., Alexander M.R., Yang J., Roberts C.J. (2015). 3D printing of five-in-one dose combination polypill with defined immediate and sustained release profiles. J. Control. Release.

[127] Shaqour B., Samaro A., Verleije B., Beyers K., Vervaet C., Cos P. (2020). Production of Drug Delivery Systems Using Fused Filament Fabrication: A Systematic Review. Pharmaceutics.

[128] Azad M.A., Olawuni D., Kimbell G., Badruddoza A.Z.M., Hossain M.S., Sultana T. (2020). Polymers for Extrusion-Based 3D Printing of Pharmaceuticals: A Holistic Materials-Process Perspective. Pharmaceutics.

[129] Tagami T., Kuwata E., Sakai N., Ozeki T. (2019). Drug Incorporation into Polymer Filament Using Simple Soaking Method for Tablet Preparation Using Fused Deposition Modeling. Biol. Pharm. Bull..

[130] Okafor-Muo O.L., Hassanin H., Kayyali R., ElShaer A. (2020). 3D Printing of Solid Oral Dosage Forms: Numerous Challenges With Unique Opportunities. J. Pharm. Sci..

[131] Skowyra J., Pietrzak K., Alhnan M.A. (2015). Fabrication of extended-release patient-tailored prednisolone tablets via fused deposition modelling (FDM) 3D printing. Eur. J. Pharm. Sci..

[132] Goyanes A., Buanz A.B., Basit A.W., Gaisford S. (2014). Fused-filament 3D printing (3DP) for fabrication of tablets. Int. J. Pharm..

[133] Wei C., Solanki N.G., Vasoya J.M., Shah A.V., Serajuddin A.T. (2020). Development of 3D printed tablets by fused deposition modeling using polyvinyl alcohol as polymeric matrix for rapid drug release. J. Pharm. Sci..

[134] Farto-Vaamonde X., Auriemma G., Aquino R.P., Concheiro A., Alvarez-Lorenzo C. (2019). Post-manufacture loading of filaments and 3D printed PLA scaffolds with prednisolone and dexamethasone for tissue regeneration applications. Eur. J. Pharm. Biopharm..

[135] Okwuosa T.C., Pereira B.C., Arafat B., Cieszynska M., Isreb A., Alhnan M.A. (2017). Fabricating a Shell-Core Delayed Release Tablet Using Dual FDM 3D Printing for Patient-Centred Therapy. Pharm. Res..

[136] Viidik L., Vesala J., Laitinen R., Korhonen O., Ketolainen J., Aruväli J., Kirsimäe K., Kogermann K., Heinämäki J., Laidmäe I. (2021). Preparation and characterization of hot-melt extruded polycaprolactone-based filaments intended for 3D-printing of tablets. Eur. J. Pharm. Sci..

[137] Long J., Gholizadeh H., Lu J., Bunt C., Seyfoddin A. (2017). Application of fused deposition modelling (FDM) method of 3D printing in drug delivery. Curr. Pharm. Des..

[138] Sandler N., Salmela I., Fallarero A., Rosling A., Khajeheian M., Kolakovic R., Genina N., Nyman J., Vuorela P. (2014). Towards fabrication of 3D printed medical devices to prevent biofilm formation. Int. J. Pharm..

[139] Domínguez-Robles J., Mancinelli C., Mancuso E., García-Romero I., Gilmore B.F., Casettari L., Larrañeta E., Lamprou D.A. (2020). 3D Printing of Drug-Loaded Thermoplastic Polyurethane Meshes: A Potential Material for Soft Tissue Reinforcement in Vaginal Surgery. Pharmaceutics.

[140] Genina N., Holländer J., Jukarainen H., Mäkilä E., Salonen J., Sandler N. (2016). Ethylene vinyl acetate (EVA) as a new drug carrier for 3D printed medical drug delivery devices. Eur. J. Pharm. Sci..

[141] Isreb A., Baj K., Wojsz M., Isreb M., Peak M., Alhnan M.A. (2019). 3D printed oral theophylline doses with innovative ‘radiator-like’design: Impact of polyethylene oxide (PEO) molecular weight. Int. J. Pharm..

[142] Dos Santos J., da Silva G.S., Velho M.C., Beck R.C.R. (2021). Eudragit^®^: A Versatile Family of Polymers for Hot Melt Extrusion and 3D Printing Processes in Pharmaceutics. Pharmaceutics.

[143] Pereira G.G., Figueiredo S., Fernandes A.I., Pinto J.F. (2020). Polymer Selection for Hot-Melt Extrusion Coupled to Fused Deposition Modelling in Pharmaceutics. Pharmaceutics.

[144] Melocchi A., Parietti F., Maroni A., Foppoli A., Gazzaniga A., Zema L. (2016). Hot-melt extruded filaments based on pharmaceutical grade polymers for 3D printing by fused deposition modeling. Int. J. Pharm..

[145] Chai X., Chai H., Wang X., Yang J., Li J., Zhao Y., Cai W., Tao T., Xiang X. (2017). Fused deposition modeling (FDM) 3D printed tablets for intragastric floating delivery of domperidone. Sci. Rep..

[146] Than Y.M., Suriyarak S., Titapiwatanakun V. (2022). Rheological Investigation of Hydroxypropyl Cellulose–Based Filaments for Material Extrusion 3D Printing. Polymers.

[147] Zhang J., Feng X., Patil H., Tiwari R.V., Repka M.A. (2017). Coupling 3D printing with hot-melt extrusion to produce controlled-release tablets. Int. J. Pharm..

[148] Konta A.A., García-Piña M., Serrano D.R. (2017). Personalised 3D Printed Medicines: Which Techniques and Polymers Are More Successful?. Bioengineering.

[149] Ibrahim M., Barnes M., McMillin R., Cook D.W., Smith S., Halquist M., Wijesinghe D., Roper T.D. (2019). 3D printing of metformin HCl PVA tablets by fused deposition modeling: Drug loading, tablet design, and dissolution studies. AAPS PharmSciTech.

[150] Xu X., Zhao J., Wang M., Wang L., Yang J. (2019). 3D Printed Polyvinyl Alcohol Tablets with Multiple Release Profiles. Sci. Rep..

[151] Tagami T., Nagata N., Hayashi N., Ogawa E., Fukushige K., Sakai N., Ozeki T. (2018). Defined drug release from 3D-printed composite tablets consisting of drug-loaded polyvinylalcohol and a water-soluble or water-insoluble polymer filler. Int. J. Pharm..

[152] Muppalaneni S., Omidian H. (2013). Polyvinyl alcohol in medicine and pharmacy: A perspective. J. Dev. Drugs.

[153] Gaaz T.S., Sulong A.B., Akhtar M.N., Kadhum A.A.H., Mohamad A.B., Al-Amiery A.A. (2015). Properties and applications of polyvinyl alcohol, halloysite nanotubes and their nanocomposites. Molecules.

[154] Saviano M., Aquino R.P., Del Gaudio P., Sansone F., Russo P. (2019). Poly (vinyl alcohol) 3D printed tablets: The effect of polymer particle size on drug loading and process efficiency. Int. J. Pharm..

[155] Goyanes A., Wang J., Buanz A., Martínez-Pacheco R., Telford R., Gaisford S., Basit A.W. (2015). 3D printing of medicines: Engineering novel oral devices with unique design and drug release characteristics. Mol. Pharm..

[156] Goyanes A., Chang H., Sedough D., Hatton G.B., Wang J., Buanz A., Gaisford S., Basit A.W. (2015). Fabrication of controlled-release budesonide tablets via desktop (FDM) 3D printing. Int. J. Pharm..

[157] Goyanes A., Fina F., Martorana A., Sedough D., Gaisford S., Basit A.W. (2017). Development of modified release 3D printed tablets (printlets) with pharmaceutical excipients using additive manufacturing. Int. J. Pharm..

[158] Verstraete G., Samaro A., Grymonpré W., Vanhoorne V., Van Snick B., Boone M., Hellemans T., Van Hoorebeke L., Remon J.P., Vervaet C. (2018). 3D printing of high drug loaded dosage forms using thermoplastic polyurethanes. Int. J. Pharm..

[159] Pereira B.C., Isreb A., Forbes R.T., Dores F., Habashy R., Petit J.-B., Alhnan M.A., Oga E.F. (2019). ‘Temporary Plasticiser’: A novel solution to fabricate 3D printed patient-centred cardiovascular ‘Polypill’architectures. Eur. J. Pharm. Biopharm..

[160] Murphy S., Leeke G., Jenkins M. (2012). A Comparison of the use of FTIR spectroscopy with DSC in the characterisation of melting and crystallisation in polycaprolactone. J. Therm. Anal. Calorim..

[161] Lee J.-H., Baik J.-M., Yu Y.-S., Kim J.H., Ahn C.B., Son K.H., Kim J.-H., Choi E.S., Lee J.W. (2020). Development of a heat labile antibiotic eluting 3D printed scaffold for the treatment of osteomyelitis. Sci. Rep..

[162] Costa P.F., Puga A.M., Díaz-Gomez L., Concheiro A., Busch D.H., Alvarez-Lorenzo C. (2015). Additive manufacturing of scaffolds with dexamethasone controlled release for enhanced bone regeneration. Int. J. Pharm..

[163] Tappa K., Jammalamadaka U., Ballard D.H., Bruno T., Israel M.R., Vemula H., Meacham J.M., Mills D.K., Woodard P.K., Weisman J.A. (2017). Medication eluting devices for the field of OBGYN (MEDOBGYN): 3D printed biodegradable hormone eluting constructs, a proof of concept study. PLoS ONE.

[164] Holländer J., Genina N., Jukarainen H., Khajeheian M., Rosling A., Mäkilä E., Sandler N. (2016). Three-dimensional printed PCL-based implantable prototypes of medical devices for controlled drug delivery. J. Pharm. Sci..

[165] Muwaffak Z., Goyanes A., Clark V., Basit A.W., Hilton S.T., Gaisford S. (2017). Patient-specific 3D scanned and 3D printed antimicrobial polycaprolactone wound dressings. Int. J. Pharm..

[166] Guerra A.J., Ciurana J. (2018). 3D-printed bioabsordable polycaprolactone stent: The effect of process parameters on its physical features. Mater. Des..

[167] Weisman J.A., Jammalamadaka U., Tappa K., Mills D.K. (2017). Doped halloysite nanotubes for use in the 3D printing of medical devices. Bioengineering.

[168] Boyle B.M., Xiong P.T., Mensch T.E., Werder T.J., Miyake G.M. (2019). 3D printing using powder melt extrusion. Addit. Manuf..

[169] Fanous M., Gold S., Muller S., Hirsch S., Ogorka J., Imanidis G. (2020). Simplification of fused deposition modeling 3D-printing paradigm: Feasibility of 1-step direct powder printing for immediate release dosage form production. Int. J. Pharm..

[170] Goyanes A., Allahham N., Trenfield S.J., Stoyanov E., Gaisford S., Basit A.W. (2019). Direct powder extrusion 3D printing: Fabrication of drug products using a novel single-step process. Int. J. Pharm..

[171] Öblom H., Zhang J., Pimparade M., Speer I., Preis M., Repka M., Sandler N. (2019). 3D-printed isoniazid tablets for the treatment and prevention of tuberculosis—Personalized dosing and drug release. AAPS PharmSciTech.

[172] Goyanes A., Kobayashi M., Martínez-Pacheco R., Gaisford S., Basit A.W. (2016). Fused-filament 3D printing of drug products: Microstructure analysis and drug release characteristics of PVA-based caplets. Int. J. Pharm..

[173] Tabriz A.G., Nandi U., Hurt A.P., Hui H.-W., Karki S., Gong Y., Kumar S., Douroumis D. (2021). 3D printed bilayer tablet with dual controlled drug release for tuberculosis treatment. Int. J. Pharm..

[174] Pietrzak K., Isreb A., Alhnan M.A. (2015). A flexible-dose dispenser for immediate and extended release 3D printed tablets. Eur. J. Pharm. Biopharm..

[175] Gioumouxouzis C.I., Baklavaridis A., Katsamenis O.L., Markopoulou C.K., Bouropoulos N., Tzetzis D., Fatouros D.G. (2018). A 3D printed bilayer oral solid dosage form combining metformin for prolonged and glimepiride for immediate drug delivery. Eur. J. Pharm. Sci..

[176] Okwuosa T.C., Stefaniak D., Arafat B., Isreb A., Wan K.-W., Alhnan M.A. (2016). A lower temperature FDM 3D printing for the manufacture of patient-specific immediate release tablets. Pharm. Res..

[177] Nukala P.K., Palekar S., Patki M., Patel K. (2019). Abuse Deterrent Immediate Release Egg-Shaped Tablet (Egglets)Using 3D Printing Technology: Quality by Design to Optimize Drug Release and Extraction. AAPS PharmSciTech.

[178] Sadia M., Sośnicka A., Arafat B., Isreb A., Ahmed W., Kelarakis A., Alhnan M.A. (2016). Adaptation of pharmaceutical excipients to FDM 3D printing for the fabrication of patient-tailored immediate release tablets. Int. J. Pharm..

[179] Melocchi A., Parietti F., Loreti G., Maroni A., Gazzaniga A., Zema L. (2015). 3D printing by fused deposition modeling (FDM) of a swellable/erodible capsular device for oral pulsatile release of drugs. J. Drug Deliv. Sci. Technol..

[180] Scoutaris N., Ross S.A., Douroumis D. (2018). 3D printed “Starmix” drug loaded dosage forms for paediatric applications. Pharm. Res..

[181] Eleftheriadis G.K., Ritzoulis C., Bouropoulos N., Tzetzis D., Andreadis D.A., Boetker J., Rantanen J., Fatouros D.G. (2019). Unidirectional drug release from 3D printed mucoadhesive buccal films using FDM technology: In vitro and ex vivo evaluation. Eur. J. Pharm. Biopharm..

[182] Vidakis N., Petousis M., Velidakis E., Mountakis N., Tzounis L., Liebscher M., Grammatikos S.A. (2021). Enhanced Mechanical, Thermal and Antimicrobial Properties of Additively Manufactured Polylactic Acid with Optimized Nano Silica Content. Nanomaterials.

[183] Seoane-Viaño I., Januskaite P., Alvarez-Lorenzo C., Basit A.W., Goyanes A. (2021). Semi-solid extrusion 3D printing in drug delivery and biomedicine: Personalised solutions for healthcare challenges. J. Control. Release.

[184] Li Q., Guan X., Cui M., Zhu Z., Chen K., Wen H., Jia D., Hou J., Xu W., Yang X. (2018). Preparation and investigation of novel gastro-floating tablets with 3D extrusion-based printing. Int. J. Pharm..

[185] Eduardo D.-T., Ana S.-E., José B.F. (2021). A micro-extrusion 3D printing platform for fabrication of orodispersible printlets for pediatric use. Int. J. Pharm..

[186] Conceição J., Farto-Vaamonde X., Goyanes A., Adeoye O., Concheiro A., Cabral-Marques H., Sousa Lobo J.M., Alvarez-Lorenzo C. (2019). Hydroxypropyl-β-cyclodextrin-based fast dissolving carbamazepine printlets prepared by semisolid extrusion 3D printing. Carbohydr. Polym..

[187] Goyanes A., Madla C.M., Umerji A., Piñeiro G.D., Montero J.M.G., Diaz M.J.L., Barcia M.G., Taherali F., Sánchez-Pintos P., Couce M.-L. (2019). Automated therapy preparation of isoleucine formulations using 3D printing for the treatment of MSUD: First single-centre, prospective, crossover study in patients. Int. J. Pharm..

[188] Tagami T., Ito E., Kida R., Hirose K., Noda T., Ozeki T. (2021). 3D printing of gummy drug formulations composed of gelatin and an HPMC-based hydrogel for pediatric use. Int. J. Pharm..

[189] Herrada-Manchón H., Rodríguez-González D., Fernández M.A., Suñé-Pou M., Pérez-Lozano P., García-Montoya E., Aguilar E. (2020). 3D printed gummies: Personalized drug dosage in a safe and appealing way. Int. J. Pharm..

[190] Rycerz K., Stepien K.A., Czapiewska M., Arafat B.T., Habashy R., Isreb A., Peak M., Alhnan M.A. (2019). Embedded 3D printing of novel bespoke soft dosage form concept for pediatrics. Pharmaceutics.

[191] Johannesson J., Khan J., Hubert M., Teleki A., Bergström C.A. (2021). 3D-printing of solid lipid tablets from emulsion gels. Int. J. Pharm..

[192] Visser J.C., Wibier L., Mekhaeil M., Woerdenbag H.J., Taxis K. (2020). Orodispersible films as a personalized dosage form for nursing home residents, an exploratory study. Int. J. Clin. Pharm..

[193] Borges A.F., Silva C., Coelho J.F., Simões S. (2015). Oral films: Current status and future perspectives: I—galenical development and quality attributes. J. Control. Release.

[194] Falcone G., Saviano M., Aquino R.P., Del Gaudio P., Russo P. (2021). Coaxial semi-solid extrusion and ionotropic alginate gelation: A successful duo for personalized floating formulations via 3D printing. Carbohydr. Polym..

[195] Naseri E., Butler H., MacNevin W., Ahmed M., Ahmadi A. (2020). Low-temperature solvent-based 3D printing of PLGA: A parametric printability study. Drug Dev. Ind. Pharm..

[196] Chou P.-Y., Chou Y.-C., Lai Y.-H., Lin Y.-T., Lu C.-J., Liu S.-J. (2021). Fabrication of drug-eluting nano-hydroxylapatite filled polycaprolactone nanocomposites using solution-extrusion 3D printing technique. Polymers.

[197] Haring A.P., Tong Y., Halper J., Johnson B.N. (2018). Programming of multicomponent temporal release profiles in 3D printed polypills via core–shell, multilayer, and gradient concentration profiles. Adv. Healthc. Mater..

[198] Siyawamwaya M., du Toit L.C., Kumar P., Choonara Y.E., Kondiah P.P., Pillay V. (2019). 3D printed, controlled release, tritherapeutic tablet matrix for advanced anti-HIV-1 drug delivery. Eur. J. Pharm. Biopharm..

[199] Goh W.J., Tan S.X., Pastorin G., Ho P.C.L., Hu J., Lim S.H. (2021). 3D printing of four-in-one oral polypill with multiple release profiles for personalized delivery of caffeine and vitamin B analogues. Int. J. Pharm..

[200] El Aita I., Rahman J., Breitkreutz J., Quodbach J. (2020). 3D-Printing with precise layer-wise dose adjustments for paediatric use via pressure-assisted microsyringe printing. Eur. J. Pharm. Biopharm..

[201] Zheng Z., Lv J., Yang W., Pi X., Lin W., Lin Z., Zhang W., Pang J., Zeng Y., Lv Z. (2020). Preparation and application of subdivided tablets using 3D printing for precise hospital dispensing. Eur. J. Pharm. Sci..

[202] Yang Y., Wang X., Lin X., Xie L., Ivone R., Shen J., Yang G. (2020). A tunable extruded 3D printing platform using thermo-sensitive pastes. Int. J. Pharm..

[203] Aita I.E., Breitkreutz J., Quodbach J. (2020). Investigation of semi-solid formulations for 3D printing of drugs after prolonged storage to mimic real-life applications. Eur. J. Pharm. Sci..

[204] Wen H., He B., Wang H., Chen F., Li P., Cui M., Li Q., Pan W., Yang X. (2019). Structure-based gastro-retentive and controlled-release drug delivery with novel 3D printing. AAPS PharmSciTech.

[205] Sjöholm E., Mathiyalagan R., Rajan Prakash D., Lindfors L., Wang Q., Wang X., Ojala S., Sandler N. (2020). 3D-Printed Veterinary Dosage Forms—A Comparative Study of Three Semi-Solid Extrusion 3D Printers. Pharmaceutics.

[206] Vithani K., Goyanes A., Jannin V., Basit A.W., Gaisford S., Boyd B.J. (2019). A proof of concept for 3D printing of solid lipid-based formulations of poorly water-soluble drugs to control formulation dispersion kinetics. Pharm. Res..

[207] Seoane-Viaño I., Ong J.J., Luzardo-Álvarez A., González-Barcia M., Basit A.W., Otero-Espinar F.J., Goyanes A. (2021). 3D printed tacrolimus suppositories for the treatment of ulcerative colitis. Asian J. Pharm. Sci..

[208] Liu J., Tagami T., Ozeki T. (2020). Fabrication of 3D-printed fish-gelatin-based polymer hydrogel patches for local delivery of pegylated liposomal doxorubicin. Mar. Drugs.

[209] Long J., Etxeberria A.E., Nand A.V., Bunt C.R., Ray S., Seyfoddin A. (2019). A 3D printed chitosan-pectin hydrogel wound dressing for lidocaine hydrochloride delivery. Mater. Sci. Eng. C.

[210] Yan J., Wang Y., Zhang X., Zhao X., Ma J., Pu X., Wang Y., Ran F., Wang Y., Leng F. (2019). Snakegourd root/Astragalus polysaccharide hydrogel preparation and application in 3D printing. Int. J. Biol. Macromol..

[211] Andriotis E.G., Eleftheriadis G.K., Karavasili C., Fatouros D.G. (2020). Development of bio-active patches based on pectin for the treatment of ulcers and wounds using 3D-bioprinting technology. Pharmaceutics.

[212] Wu M., Zhang Y., Huang H., Li J., Liu H., Guo Z., Xue L., Liu S., Lei Y. (2020). Assisted 3D printing of microneedle patches for minimally invasive glucose control in diabetes. Mater. Sci. Eng. C.

[213] Deng N., Sun J., Li Y., Chen L., Chen C., Wu Y., Wang Z., Li L. (2019). Experimental study of rhBMP-2 chitosan nano-sustained release carrier-loaded PLGA/nHA scaffolds to construct mandibular tissue-engineered bone. Arch. Oral Biol..

[214] Lin H.Y., Chang T.W., Peng T.K. (2018). Three-dimensional plotted alginate fibers embedded with diclofenac and bone cells coated with chitosan for bone regeneration during inflammation. J. Biomed. Mater. Res. Part A.

[215] Marques C.F., Olhero S.M., Torres P.M., Abrantes J.C., Fateixa S., Nogueira H.I., Ribeiro I.A., Bettencourt A., Sousa A., Granja P.L. (2019). Novel sintering-free scaffolds obtained by additive manufacturing for concurrent bone regeneration and drug delivery: Proof of concept. Mater. Sci. Eng. C.

[216] Etxabide A., Long J., Guerrero P., de la Caba K., Seyfoddin A. (2019). 3D printed lactose-crosslinked gelatin scaffolds as a drug delivery system for dexamethasone. Eur. Polym. J..

[217] Borandeh S., van Bochove B., Teotia A., Seppälä J. (2021). Polymeric drug delivery systems by additive manufacturing. Adv. Drug Deliv. Rev..

[218] Yang Y., Wang H., Li H., Ou Z., Yang G. (2018). 3D printed tablets with internal scaffold structure using ethyl cellulose to achieve sustained ibuprofen release. Eur. J. Pharm. Sci..

[219] Gioumouxouzis C.I., Tzimtzimis E., Katsamenis O.L., Dourou A., Markopoulou C., Bouropoulos N., Tzetzis D., Fatouros D.G. (2020). Fabrication of an osmotic 3D printed solid dosage form for controlled release of active pharmaceutical ingredients. Eur. J. Pharm. Sci..

[220] Thanawuth K., Sutthapitaksakul L., Konthong S., Suttiruengwong S., Huanbutta K., Dass C.R., Sriamornsak P. (2021). Impact of Drug Loading Method on Drug Release from 3D-Printed Tablets Made from Filaments Fabricated by Hot-Melt Extrusion and Impregnation Processes. Pharmaceutics.

[221] Sun Y., Soh S. (2015). Printing Tablets with Fully Customizable Release Profiles for Personalized Medicine. Adv. Mater..

[222] Kadry H., Al-Hilal T.A., Keshavarz A., Alam F., Xu C., Joy A., Ahsan F. (2018). Multi-purposable filaments of HPMC for 3D printing of medications with tailored drug release and timed-absorption. Int. J. Pharm..

[223] Pereira B.C., Isreb A., Isreb M., Forbes R.T., Oga E.F., Alhnan M.A. (2020). Additive Manufacturing of a Point-of-Care “Polypill:” Fabrication of Concept Capsules of Complex Geometry with Bespoke Release against Cardiovascular Disease. Adv. Healthc. Mater..

[224] Melocchi A., Uboldi M., Maroni A., Foppoli A., Palugan L., Zema L., Gazzaniga A. (2020). 3D printing by fused deposition modeling of single- and multi-compartment hollow systems for oral delivery-A review. Int. J. Pharm..

[225] Melocchi A., Parietti F., Maccagnan S., Ortenzi M.A., Antenucci S., Briatico-Vangosa F., Maroni A., Gazzaniga A., Zema L. (2018). Industrial Development of a 3D-Printed Nutraceutical Delivery Platform in the Form of a Multicompartment HPC Capsule. AAPS PharmSciTech.

[226] Tan Y.J.N., Yong W.P., Kochhar J.S., Khanolkar J., Yao X., Sun Y., Ao C.K., Soh S. (2020). On-demand fully customizable drug tablets via 3D printing technology for personalized medicine. J. Control. Release.

[227] Trenfield S.J., Awad A., Madla C.M., Hatton G.B., Firth J., Goyanes A., Gaisford S., Basit A.W. (2019). Shaping the future: Recent advances of 3D printing in drug delivery and healthcare. Expert. Opin. Drug. Deliv..

